# Effect of selenium supplementation on musculoskeletal health in older women: a randomised, double-blind, placebo-controlled trial

**DOI:** 10.1016/S2666-7568(21)00051-9

**Published:** 2021-04

**Authors:** Jennifer S Walsh, Richard M Jacques, Lutz Schomburg, Tom R Hill, John C Mathers, Graham R Williams, Richard Eastell

**Affiliations:** a Mellanby Centre for Musculoskeletal Research, University of Sheffield, Northern General Hospital, Sheffield, UK; b School of Health and Related Research, University of Sheffield, Sheffield, UK; c Institut für Experimentelle Endokrinologie, Campus Virchow-Klinikum, Berlin, Germany; d Human Nutrition Research Centre, Centre for Healthier Lives, Population Health Science Institute, Newcastle University, Newcastle upon Tyne, UK; e Molecular Endocrinology Laboratory, Department of Metabolism, Digestion and Reproduction, Imperial College London, London, UK

## Abstract

**Background:**

Observational and preclinical studies show associations between selenium status, bone health, and physical function. Most adults in Europe have serum selenium below the optimum range. We hypothesised that selenium supplementation could reduce pro-resorptive actions of reactive oxygen species on osteoclasts and improve physical function.

**Methods:**

We completed a 6-month randomised, double-blind, placebo-controlled trial. We recruited postmenopausal women older than 55 years with osteopenia or osteoporosis at the Northern General Hospital, Sheffield, UK. Participants were randomly assigned 1:1:1 to receive selenite 200 μg, 50 μg, or placebo orally once per day. Medication was supplied to the site blinded and numbered by a block randomisation sequence with a block size of 18, and participants were allocated medication in numerical order. All participants and study team were masked to treatment allocation. The primary endpoint was urine N-terminal cross-linking telopeptide of type I collagen (NTx, expressed as ratio to creatinine) at 26 weeks. Analysis included all randomly assigned participants who completed follow-up. Groups were compared with analysis of covariance with Hochberg testing. Secondary endpoints were other biochemical markers of bone turnover, bone mineral density, short physical performance battery, and grip strength. Mechanistic endpoints were glutathione peroxidase, highly sensitive C-reactive protein, and interleukin-6. This trial is registered with EU clinical trials, EudraCT 2016-002964-15, and ClinicalTrials.gov, NCT02832648, and is complete.

**Findings:**

120 participants were recruited between Jan 23, 2017, and April 11, 2018, and randomly assigned to selenite 200 μg, 50 μg, or placebo (n=40 per group). 115 (96%) of 120 participants completed follow-up and were included in the primary analysis (200 μg [n=39], 50 μg [n=39], placebo [n=37]). Median follow-up was 25·0 weeks (IQR 24·7–26·0). In the 200 μg group, mean serum selenium increased from 78·8 (95% CI 73·5–84·2) to 105·7 μg/L (99·5–111·9). Urine NTx to creatinine ratio (nmol bone collagen equivalent:mmol creatinine) did not differ significantly between treatment groups at 26 weeks: 40·5 (95% CI 34·9–47·0) for placebo, 43·4 (37·4–50·5) for 50 μg, and 42·2 (37·5–47·6) for 200 μg. None of the secondary or mechanistic endpoint measurements differed between treatment groups at 26 weeks. Seven (6%) of 120 participants were withdrawn from treatment at week 13 due to abnormal thyroid-stimulating hormone concentrations (one in the 200 μg group, three in the 50 μg group, and three in the placebo group) and abnormal blood glucose (one in the 50 μg group). There were three serious adverse events: a non-ST elevation myocardial infarction at week 18 (in the 50 μg group), a diagnosis of bowel cancer after routine population screening at week 2 (in the placebo group), and a pulmonary embolus due to metastatic bowel cancer at week 4 (in the 200 μg group). All severe adverse events were judged by the principal investigator as unrelated to trial medication.

**Interpretation:**

Selenium supplementation at these doses does not affect musculoskeletal health in postmenopausal women.

**Funding:**

UK National Institute for Health Research Efficacy and Mechanism Evaluation programme.

## Introduction

One in two women and one in five men older than 50 years in the UK will have a fragility fracture, defined as a fracture that results from a fall from standing height or less. About 30% of women older than 65 years are osteopenic and at risk of developing osteoporosis and fractures. More than half of all fractures in postmenopausal women occur in women with osteopenia. Women with osteopenia are generally not given osteoporosis treatment because of the individual risk to benefit ratio. Previously, these women would have been offered hormone replacement therapy for bone protection, but adverse effects of this therapy have limited its use. Bisphosphonates are the mainstay of osteoporosis treatment for women at higher risk, but use of these medications has declined due to physician and patient wariness of adverse events.^1^ Calcium and vitamin D supplements are generally recommended for osteopenic adults, but the bone density benefits are small. Therefore, there is a need for an effective, safe, well tolerated, inexpensive, and widely applicable treatment option for osteopenic women.

**Research in context**

**Evidence before this study**

We searched PubMed and Google Scholar. Search terms were “selenium”, “bone”, “osteoporosis”, “fracture”, “physical function”, “muscle”, and “mortality”. The search was limited to English language publications and was not limited by publication date. Preclinical, epidemiological, and observational studies suggest a role for selenium in bone and muscle health, and analysis of the Osteoporosis and Ultrasound (OPUS) study data by some of the authors of this paper confirmed a relationship between serum selenium, bone turnover markers, and bone mineral density. Clinical trials have shown efficacy of selenium supplements for cancer prevention and Graves' eye disease.

**Added value of this study**

This study is the first randomised, double-blind placebo-controlled trial of selenium for musculoskeletal health. Supplemental sodium selenite (200 μg or 50 μg daily) for 6 months had no effect on biochemical markers of bone turnover or physical function tests.

**Implications of all the available evidence**

Selenium supplementation at these doses does not affect bone health or physical function in postmenopausal women. Observed associations between selenium status and bone health could be influenced by other factors. A single nutrient supplementation approach with selenium might be ineffective in this context.

High bone turnover is the principal mechanism of osteoporotic bone loss, and inflammatory cytokines and reactive oxygen species are potent stimuli for bone resorption.^2^, ^3^ An increase in reactive oxygen species has been proposed as a key mechanism by which sex hormone deficiency causes age-related bone loss through the RANK pathway.^4^

In 1144 postmenopausal women older than 55 years from the UK, France, and Germany, higher serum selenium or selenoprotein P was associated with higher bone mineral density (BMD) of the lumbar spine and total hip, and lower biochemical markers of bone turnover.^5^ Serum selenium concentration was also associated with balance and grip strength. Lower serum selenium was associated with higher free T4 and T3, but the associations of selenium with bone measures were independent of thyroid hormones.

Selenoproteins contribute to anti-inflammatory and antioxidative pathways, and increased selenoprotein expression is associated with reduced interleukin (IL)-6 and reactive oxygen species,^6^, ^7^ and so it is plausible that selenium could affect bone metabolism directly. Selenoproteins are expressed in osteoblasts and osteoclasts, and are found in the bone microenvironment.^8^, ^9^ Through antioxidative catalysis and reducing reactive oxygen species, selenium could directly antagonise a key cellular mechanism in the pathogenesis of postmenopausal osteoporosis.

There is animal evidence to support the hypothesis that selenium has a role in bone biology and decreases bone turnover. Selenium deficient rodents have abnormal bone growth, poorer bone microarchitecture, higher bone resorption markers, and higher inflammatory markers than selenium replete controls.^10^, ^11^

Endemic selenium deficiency in humans has been associated with the osteoarthropathy Kashin-Beck disease.^12^ Selenium status has also been associated with BMD in men in the Netherlands,^13^ and higher selenium intake was associated with lower hip fracture risk in adults older than 50 years in the USA.^14^

Selenium is obtained from dietary intake; the main sources of selenium in the UK are bread, cereals, seafood, and meat. The main determinant of food selenium content is soil selenium availability. The recommended adequate intake for adults older than 50 years in the UK is 75 μg for men and 60 μg for women, daily, but the mean intake is only 40 μg daily.^15^ The decreasing intake in the UK is due to a shift in the source of flour for bread-making from North America (which contains more selenium) to Europe, changes in fertiliser practice, and reduced industrial emissions.^16^

Studies of all-cause mortality suggest that the optimum range of serum selenium for human health is approximately 120–150 μg/L. Most adults in the UK have serum selenium between 80 and 100 μg/L.^15^

Several other age-related disorders are linked to inadequate selenium status, including cardiovascular disease, poor cognitive function, and reduced muscle strength.^17^, ^18^ Selenium supplementation (200 μg daily as selenised yeast) with enzyme Q10 reduced cardiovascular mortality and markers of inflammation and increased IGF-1 in Swedish adults older than 70 years.^19^, ^20^ In the Nutritional Prevention of Cancer trial, selenium supplementation (also 200 μg daily as selenised yeast) reduced all-cancer risk in people with lower baseline serum selenium^21^ and meta-analyses generally find a beneficial effect of selenium on cancer risk.^15^

Possible adverse effects of selenium supplementation include increased risk of type 2 diabetes. However, this was not seen when the large randomised supplementation studies were systematically evaluated by meta-analysis.^22^

We hypothesised that in a relatively selenium deficient population such as that in the UK, selenium supplementation would decrease bone turnover by increasing selenoprotein expression and activity, reducing osteoclast activity, and might improve muscle function. In the longer term, both of these actions could reduce fracture risk.

The objectives of the study were to determine if selenium supplementation in postmenopausal women with osteopenia decreases bone turnover, improves physical function score and grip strength, is safe (particularly for thyroid function and diabetes), increases biomarkers of selenium status, and decreases markers of oxidative stress and inflammation.

## Methods

### Study design and participants

We conducted a randomised, double-blind, placebo-controlled study of 6 months of selenium supplementation in postmenopausal women with osteopenia or osteoporosis. This was a single centre study, at the Northern General Hospital, Sheffield, UK.

Participants were recruited from a database of volunteers, by poster and email advertising, and from patients attending the metabolic bone centre for bone densitometry. Inclusion criteria were: age older than 55 years, at least 5 years since last menstrual period, osteopenia or osteoporosis (dual-energy x-ray absorptiometry BMD lowest T-score between −1·0 and −3·0 at lumbar spine or total hip), and willing and able to give informed consent. Exclusion criteria were: diabetes, thyroid dysfunction, any conditions known to affect bone metabolism (such as inflammatory disease, parathyroid disease, malabsorption, high alcohol intake, and prolonged immobility), fracture or orthopaedic surgery in the last year, osteoporosis treatment or drugs known to affect bone metabolism in the last year, selenium supplements in the last 60 days, or previous adverse reaction to selenium or any of the selenite or placebo excipients. Women taking calcium and vitamin D supplements were not excluded if they had been taking the calcium and vitamin D for at least 60 days and planned to continue throughout the trial. We did not set inclusion and exclusion criteria based on serum selenium status, so that the results of this study could be generalised into practice. However, we specified that only women with baseline serum selenium of less than 120 μg/L would be included in the primary analysis.

All participants gave written informed consent and the study was done in accordance with the Declaration of Helsinki. The study was approved by Yorkshire and the Humber Research Ethics Committee (REC reference 16/YH/0393; appendix pp 1, 9).

### Randomisation and masking

Participants were randomly assigned 1:1:1 to either placebo or two different doses of selenium supplementation.

Sharp Clinical Services (Crickhowell, UK) generated a block randomisation sequence with a block size of 18 and no stratification. Medication containers were supplied to the site pharmacy blinded and numbered according to the randomisation sequence. Participants were allocated medication in numerical order. An emergency code break was held in the site pharmacy but was not accessed by the study team during the trial. JSW enrolled all participants. All participants and study team were blind to treatment allocation until after database lock. The selenite tablets were over-encapsulated and a matching placebo was also manufactured by Sharp Clinical Services.

### Procedures

The interventions were sodium selenite tablets 50 μg or 200 μg (Selenase, Biosyn, Fellbach, Germany) or placebo orally, once a day. We chose a daily dose of 200 μg because this dose has previously been shown to be safe and have clinically significant effects in the Nutritional Prevention of Cancer trial^21^ and in treatment of Graves' ophthalmopathy.^23^ We estimated that this dose would increase serum selenium by approximately 60 μg/L.^24^ The daily dose of 50 μg was included to assess dose response; if 50 μg and 200 μg had similar effects, we could recommend the 50 μg dose for clinical use, at lower cost and lower risk of adverse effects.

All participants were given a single oral dose of 100 000 IU colecalciferol at screening, to ensure they were vitamin D sufficient at the start of trial treatment.

Participants attended for four visits: screening, baseline with randomisation, 13 weeks, and 26 weeks. They were contacted by telephone at 6 weeks, 19 weeks, and 30 weeks. Height, weight, pulse, blood pressure, grip strength, short physical performance battery, and blood and urine tests were taken at baseline, 13 weeks, and 26 weeks. Diet diaries and dual-energy x-ray absorptiometry BMD were done at screening and 26 weeks. Participants were withdrawn from treatment if they had abnormal concentrations of thyroid-stimulating hormone, glycated haemoglobin, or blood glucose at 13 weeks, or if they had serious adverse events judged to be due to the study medication, but they were asked to continue in follow-up for the primary analysis. Participants were only removed from the trial if they withdrew consent.

### Outcomes

The primary endpoint was between-group difference in the ratio of urine N-terminal cross-linking telopeptide of type I collagen (NTx) to creatinine at 26 weeks. Bone turnover markers change much more rapidly than BMD, so can determine more quickly and cost-effectively if an intervention is likely to work. We chose NTx because the relationship between change in NTx and change in fracture risk is known. With bisphosphonate treatment,^25^ a 30% decrease in NTx is associated with a 40% reduction in spine fracture, and 66% of the vertebral fracture risk reduction at 3 years is explained by change in NTx. Also, NTx was the marker most strongly related to selenium status in the previous observational study.^5^

The secondary endpoints were: serum selenium and selenoprotein P; other bone turnover markers (procollagen type I N propeptide [PINP], osteocalcin, C-terminal cross-linking telopeptide of type I collagen [CTx]), BMD of the lumbar spine and total hip by dual-energy x-ray absorptiometry, muscle function (short physical performance battery and hand grip strength); antioxidant and inflammatory markers (glutathione peroxidase activity [a selenium-containing antioxidant that is increased in postmenopausal women with osteopenia],^26^ IL-6, and highly sensitive C-reactive protein).

Blood samples for biochemical measurements were taken fasted in the morning. Serum samples were obtained in serum separation tubes, allowed to clot for 30 min, then centrifuged at 2500 rpm for 10 min and separated into aliquots. Urine samples were obtained as triplicate samples from fasted second morning voids on three days immediately before the study visit and kept refrigerated until the study visit. Equal volume aliquots from the urine samples were pooled into a single sample, then the pooled sample was separated into aliquots. Samples were frozen at −80°C and analysed in batches at the end of the study.

Urine NTx was measured by automated immunoassay (Vitros ECiQ, Ortho Clincal Diagnostics, High Wycombe, UK) at PathLab London (interassay coefficient of variability [CV] 6%). NTx was expressed as a ratio to urinary creatinine concentration measured by the dry slide method (Vitros 250, Ortho Clinical Diagnostics, interassay CV 3%). CTx, osteocalcin, PINP, and 25-hydroxyvitamin D were measured by automated immunoassay (IDS-iSYS, Immunodiagnostic Systems, Boldon, UK), by the University of Sheffield Academic Unit of Bone Metabolism. The interassay CVs are 6·5, 5·0, 7·2, and 6·7%, respectively. Highly sensitive C-reactive protein and IL-6 were measured by automated immunoassay by the Sheffield Teaching Hospitals Clinical Immunology laboratory. Serum selenium was measured by x-ray fluorescence spectroscopy (S4 T-STAR, Bruker Nano Analytics, Berlin, Germany), selenoprotein P was measured by a validated immunoassay (selenOtest, selenOmed, Berlin, Germany), and glutathione peroxidase was measured by enzyme analysis using tButyl-OOH as substrate at the Institute for Experimental Endocrinology, Charité–University Medical School Berlin, Germany.

Height and weight were measured with an electric scale and stadiometer to the nearest 0·1 cm and 0·1 kg. Pulse and blood pressure were measured with an automated sphygmomanometer (Dinamap, GE Healthcare, Chalfont St Giles, UK). Grip strength was assessed using a digital hand dynamometer (Saehan Corporation, Masan, South Korea). Three measurements were taken for each hand and the best value used for analysis. Short physical performance battery score was calculated from a chair stand, tandem balance, and narrow walk test.

BMD was assessed by dual-energy x-ray absorptiometry of the spine and hip (Discovery, Hologic, Manchester, UK) at baseline and at 26 weeks according to standard scanning protocols, by specialist scan technicians in the Sheffield Clinical Research Facility, UK.

Dietary selenium and other nutrient intake were assessed with 7-day diet diaries. The purpose of the food diaries was to describe participants' habitual dietary intake of selenium and nutrients, which influence bone turnover. The diaries were analysed using DIETQ (Tinuviel Software, Warrington, UK) supervised by a nutritionist with experience in clinical research.

Safety assessments for diabetes and thyroid function were made at screening, baseline, 13 weeks, and 26 weeks. The measurements were made in real time by Sheffield Teaching Hospitals pathology laboratories.

Adverse events (including questioning for possible symptoms of selenium toxicity) were collected from the time of consent, at study visits, and during telephone contact throughout the treatment period and 4 weeks after the end of the treatment.

### Statistical analysis

We hypothesised that selenium supplementation would decrease bone turnover and improve physical function.

The study had 90% power to detect a 20% between-group difference at the 2·5% (two-sided) level (approximately 10 nmol bone collagen equivalent:mmol creatinine) in NTx to creatinine ratio at 26 weeks. A 20% decrease in NTx (about 1 SD decrease) with bisphosphonate treatment is associated with a 30% decrease in incident vertebral fracture.^27^ We set 20% as a plausible effect size, based on estimated change in serum selenium and the regression coefficient of serum selenium and NTx to creatinine ratio in our previous study. We did not expect as large a change in bone turnover as a potent antiresorptive drug such as a bisphosphonate, but perhaps similar to a weaker anti-resorptive such as a selective oestrogen receptor modulator. In a study of 6 months' treatment with lasofoxifene in 51 postmenopausal women,^28^ NTx to creatinine ratio decreased by 29%. We also used this study to estimate the SD (12·5) and the correlation between NTx to creatinine ratio at baseline and at 6 months (0·7). The primary analysis was analysis of covariance, and 21 patients per group were required.^29^ To allow for dropout, group imbalance, participants with baseline serum selenium of more than 120 μg/L, and the secondary endpoint analyses, we recruited 40 patients per group.

We included an interim analysis of baseline serum selenium after the first 40 participants were recruited, with a plan to increase the sample size to ensure 120 participants with baseline serum selenium of less than 120 μg/L for inclusion in the primary analysis. The only data reviewed were blinded baseline serum selenium concentrations. All the first 40 participants had baseline serum selenium of less than 120 μg/L, so we maintained the original recruitment target of 120 participants. We did a secondary analysis of all participants to determine whether baseline serum selenium was a determinant of bone turnover response.

A detailed statistical analysis plan was developed and approved by the trial steering committee before locking the trial database. We did an intention-to-treat analysis (all randomised participants) and per-protocol analysis. The per-protocol analysis included completing participants who took at least 75% of the study drug, assessed by reported missed doses and returned tablet count.

Baseline data were assessed for comparability between the treatment groups. Normality of distribution of variables was assessed from either the raw data or the residuals from the model using a density plot or histogram.

Primary endpoint analysis was the between-group difference in urine NTx to creatinine ratio at 26 weeks. Analysis of covariance was used with 26-week NTx to creatinine measurement as the dependent outcome variable and treatment group and baseline NTx to creatinine measurement as the independent variables. If the residuals from the model were not normally distributed, the values would be log transformed and the treatment group differences back transformed to be presented as a ratio.

The statistical analysis plan prespecified a Hochberg testing procedure^30^ for the primary endpoint which allows for comparison of the three treatment arms while maintaining the overall type I error rate at 5%. Significance would be declared for the comparison of placebo to selenium if, and only if, both selenium doses were significant at the 5% level or if either comparison was significant at 2·5%. If and only if significance was declared for both selenium doses, a comparison would be made between the doses. The comparison of 200 μg selenium versus 50 μg selenium would be made at the 5% level of significance.

We examined the effect of baseline selenium concentrations on the NTx to creatinine ratio response to selenium supplementation by fitting a linear model with NTx to creatinine ratio at follow-up as the dependent variable and baseline selenium, baseline NTx to creatinine ratio, and dose as independent variables.

The statistical analysis plan prespecified a multiple imputation strategy for the primary endpoint with 20 imputations using baseline and week 13 measurements of NTx to creatinine ratio, age of patient, and treatment allocation to replace missing data. The plan specified that additional variables associated with missing data would also be included in the multiple imputation model to make the missing at random assumption as plausible as possible. The nature of missingness and other baseline variables was explored in relation to missing data on the primary endpoint using univariable logistic regression models. The only baseline variable predictive of missingness was body-mass index. The final multiple imputation model therefore used baseline and week 13 measurements of NTx to creatinine ratio, age of patient, body-mass index of patient at baseline, and treatment allocation. Seven of the week 26 NTx to creatinine ratio values and two of the baseline NTx to creatinine ratio values were missing and were replaced by imputation. The results using the imputation model did not differ from the primary analysis.

Analysis of secondary endpoints included urine NTx to creatinine ratio at 13 weeks and BMD by dual-energy x-ray absorptiometry at 26 weeks, which were analysed as described for the primary endpoint. All other secondary endpoint measurements at 13 and 26 weeks were compared between treatment groups using linear mixed models with a random intercept to allow for multiple measurements on individuals.

The models included fixed factors for treatment group and post randomisation time, and a covariate for the baseline measurement of the outcome. To determine if the effect of treatment changed with time, an interaction between treatment group and time was tested. If this interaction was not statistically significant, it was removed from the model and the overall treatment difference was reported. If there was a significant difference between treatment groups, pairwise comparisons were made between each treatment group and placebo and then the two doses against each other. In the original protocol we planned to measure hydroperoxidases as a mechanistic variable, but we did not make these measurements because there was no suitable assay available when the trial was completed.

This study is registered with EU clinical trials, EudraCT 2016-002964-15, and ClinicalTrials.gov NCT02832648. The trial was supervised by a Trial Steering Committee and a Data Monitoring Committee.

### Role of the funding source

The National Institute for Health Research (NIHR) had some initial input to the study design through the grant application review process. The NIHR had no role in data collection, data analysis, or data interpretation. The full study report was subject to independent review through standard NIHR processes.

## Results

We recruited 120 women between Jan 23, 2017, and April 11, 2018. The last participant completed follow-up on Nov 6, 2018. Median follow-up was 25·0 weeks (IQR 24·7–26·0). 115 (96%) of 120 participants completed follow-up and were included in the primary analysis (figure). Baseline characteristics are shown in table 1. The groups were generally well balanced, and the mean baseline serum selenium was 79·4 μg/L (range 35·1–116·5). As all participants had baseline serum selenium below 120 μg/L, all were included in the primary analysis. Weight and body-mass index did not change over 26 weeks in any of the groups (data not shown).

**Figure:**
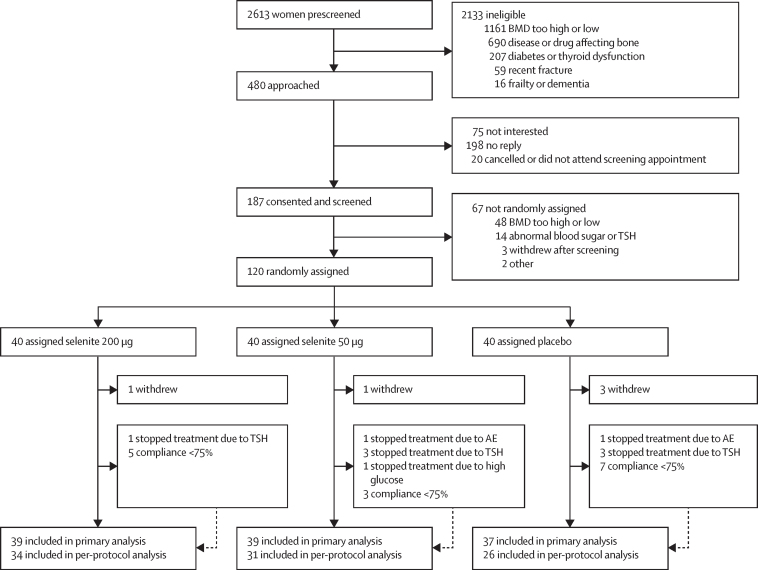
Trial profile The number of participants included in the per-protocol analysis differs from the per-protocol NTx results table (appendix p 2) due to missing NTx measurements. AE=adverse event. BMD=bone mineral density. NTx=N-terminal cross-linking telopeptide of type I collagen. TSH=thyroid-stimulating hormone.

**Table 1:** Baseline participant characteristics by treatment group

	**Placebo (n=37)**	**Selenite 50 μg (n=39)**	**Selenite 200 μg (n=39)**
**Age, years**			
n	37	39	39
Mean (SD)	66·6 (6·0)	66·7 (6·1)	64·5 (6·1)
**Height, cm**			
n	37	39	39
Mean (SD)	160·6 (5·7)	162·0 (6·4)	161·5 (7·9)
**Weight, kg**			
n	37	39	39
Mean (SD)	65·7 (11·2)	65·5 (9·2)	66·9 (10·8)
**Body-mass index, kg/m^2^**			
n	37	39	39
Mean (SD)	25·6 (4·8)	25·0 (3·7)	25·7 (3·7)
**Serum selenium, μg/L**			
n	37	38	39
Mean (SD)	80·2 (14·2)	79·3 (15·6)	78·8 (16·5)
**Serum selenoprotein P, mg/L**			
n	37	38	39
Mean (SD)	5·22 (1·45)	5·21 (1·47)	5·15 (1·37)
**Lumbar spine bone mineral density T–score**			
n	35	38	37
Mean (SD)	−1·7 (0·9)	−1·8 (1·0)	−1·8 (0·6)
**Total hip bone mineral density T–score**			
n	35	39	39
Mean (SD)	−1·3 (0·7)	−1·2 (0·7)	−0·9 (0·6)
**Urine NTx to creatinine ratio, nmol bone collagen equivalent:mmol creatinine**			
n	37	37	39
Median (IQR)	37·5 (29·7 to 49·1)	38·2 (33·7 to 49·7)	42·0 (35·0 to 49·5)
**Serum 25-hydroxyvitamin D, ng/ml**			
n	37	38	39
Mean (SD)	37·8 (10·8)	39·5 (12·1)	37·7 (12·7)

NTx=N-terminal cross-linking telopeptide of type I collagen.

The sample size calculation assumed a correlation between baseline and week 26 NTx to creatinine ratio measurement of 0·7. In the primary analysis population, the Pearson correlation between baseline and week 26 NTx to creatinine ratio was 0·62 (95% CI 0·49–0·73). The residuals from the model were not normally distributed so the NTx to creatinine ratio was log-transformed and the treatment group differences were back transformed to be presented as a ratio.

The primary endpoint (urine NTx to creatinine ratio) did not differ between treatment groups after 26 weeks or 13 weeks (table 2). 86 (72%) of 120 participants were included in the per-protocol analysis, and NTx to creatinine ratio in this group did not differ between treatment groups after 26 weeks or 13 weeks (appendix p 2).

**Table 2:** Biochemical markers of bone turnover at baseline, 13 weeks, and 26 weeks by treatment group

	**Placebo**		**Selenite 50 μg**		**Selenite 200 μg**		**Selenite 50 μg *vs* placebo**		**Selenite 200 μg *vs* placebo**		**Selenite 200 μg *vs* 50 μg**	
	n	Mean	n	Mean	n	Mean	Ratio	p value	Ratio	p value	Ratio	p value
**Urine NTx–creatinine ratio, nmol bone collagen equivalent:mmol creatinine**												
Baseline	34	37·7 (32·5–43·6)	35	40·1 (35·9–44·8)	37	41·9 (37·0–47·4)	..	..	..	..	..	..
26 weeks	34	40·5 (34·9–47·0)	35	43·4 (37·4–50·5)	37	42·2 (37·5–47·6)	1·03 (0·88–1·19)	0·74	0·97 (0·83–1·12)	0·66	0·94 (0·81–1·09)	0·43
**Urine NTx–creatinine ratio, nmol bone collagen equivalent:mmol creatinine**												
Baseline	35	37·6 (32·6–43·3)	36	40·2 (36·1–44·7)	39	42·2 (37·5–47·6)	..	..	..	..	..	..
13 weeks	35	39·7 (34·4–45·8)	36	42·0 (37·3–47·3)	39	43·1 (39·0–47·7)	1·01 (0·90–1·13)	0·88	1·00 (0·89–1·12)	0·99	0·99 (0·89–1·11)	0·89
**Serum PINP, μg/L**												
Baseline	37	48·2 (43·3–53·6)	38	50·2 (44·6–56·5)	39	49·6 (44·5–55·4)	..	..	..	..	..	..
13 weeks	36	46·1 (41·5–51·3)	37	49·9 (44·0–56·7)	39	49·6 (44·0–56·0)	..	..	..	..	..	..
26 weeks	34	47·0 (42·5–52·0)	37	46·8 (41·0–53·3)	37	47·0 (41·3–53·6)	0·97 (0·91–1·04)	0·38	0·99 (0·93–1·06)	0·82	1·02 (0·96–1·09)	0·51
**Serum osteocalcin, μg/L**												
Baseline	37	15·7 (13·4–18·4)	38	14·8 (12·7–17·2)	39	15·7 (13·8–17·9)	..	..	..	..	..	..
13 weeks	36	14·0 (12·2–16·1)	37	15·7 (13·8–17·9)	39	15·0 (13·3–17·0)	..	..	..	..	..	..
26 weeks	34	14·4 (12·6–16·4)	37	14·1 (12·3–16·2)	37	13·9 (12·4–15·6)	1·07 (0·95–1·21)	0·34	1·01 (0·89–1·15)	0·85	0·95 (0·84–1·08)	0·44
**Serum CTX, μg/L**												
Baseline	37	0·15 (0·11–0·22)	38	0·14 (0·10–0·19)	39	0·15 (0·12–0·21)	..	..	..	..	..	..
13 weeks	35	0·13 (0·10–0·17)	36	0·15 (0·11–0·21)	37	0·13 (0·10–0·18)	..	..	..	..	..	..
26 weeks	34	0·13 (0·09–0·17)	37	0·12 (0·09–0·16)	37	0·11 (0·09–0·15)	1·07 (0·80–1·45)	0·66	0·97 (0·72–1·30)	0·81	0·90 (0·67–1·20)	0·47

Data are n, mean (95% CI), ratio of means (95% CI), or p value. All urine NTx–creatinine ratios of means for treatment group from ANCOVA model include adjusting for baseline measurement. All serum ratios of means for treatment group from mixed effects model include adjusting for baseline measurement and after treatment time. Interactions between treatment group and post treatment time were not statistically significant (p>0·050) so estimates are for overall post treatment differences. Outcome variables were log transformed and the treatment group difference back transformed–give a ratio. NTx=N-terminal cross-linking telopeptide of type I collagen. PINP=procollagen type I N propeptide. CTx=C-terminal cross-linking telopeptide of type I collagen.

Mean serum selenium and selenoprotein P increased from baseline to 26 weeks in the treatment groups and there was no change in the placebo group (table 3 and appendix p 3).

**Table 3:** Serum selenium and selenoprotein P at 13 weeks and 26 weeks by treatment group

	**Placebo**		**Selenite 50 μg**		**Selenite 200 μg**		**Selenite 50 μg *vs* placebo**		**Selenite 200 μg *vs* placebo**		**Selenite 200 μg *vs* 50 μg**	
	n	Mean	n	Mean	n	Mean	Difference	p value	Difference	p value	Difference	p value
**Serum selenium, μg/L**												
Baseline	37	80·2 (75·5 to 85·0)	38	79·3 (74·2 to 84·4)	39	78·8 (73·5 to 84·2)	..	..	..	..	..	..
13 weeks	37	81·7 (77·1 to 86·4)	37	104·1 (98·5 to 109·7)	38	107·9 (102·3 to 113·4)	..	..	..	..	..	..
26 weeks	33	77·7 (73·3 to 82·2)	37	96·2 (90·7 to 101·6)	39	105·7 (99·5 to 111·9)	20·5 (14·5 to 26·5)	<0·0001	27·5 (21·6 to 33·4)	<0·0001	7·0 (1·1 to 12·8)	0·020
**Serum selenoprotein P, mg/L**												
Baseline	37	5·22 (4·73 to 5·70)	38	5·21 (4·73 to 5·70)	39	5·15 (4·71 to 5·60)	..	..	..	..	..	..
13 weeks	37	5·50 (5·11 to 5·91)	37	6·85 (6·24 to 7·46)	38	6·47 (5·89 to 7·04)	..	..	..	..	..	..
26 weeks	33	5·31 (4·75 to 5·87)	37	6·25 (5·79 to 6·70)	39	6·03 (5·54 to 6·51)	1·17 (0·62 to 1·72)	<0·0001	0·88 (0·34 to 1·42)	0·0018	−0·29 (−0·83 to 0·25)	0·29

Data are n, mean (95% CI), difference in means (95% CI), or p value. Difference in means for treatment group from linear mixed model adjusting for baseline measurement and post treatment time. Interactions between treatment group and post treatment time were not statistically significant (p>0·050) so estimates are for overall post treatment differences.

A linear regression model was fitted with log (week 26 NTx to creatinine ratio) as the dependent variable and log (baseline NTx to creatinine ratio), baseline selenium, and treatment group as independent variables. The interaction between treatment group and baseline selenium was not statistically significant (p=0·47) suggesting that treatment group did not modify the relationship between baseline selenium and 26-week NTx to creatinine ratio.

There were no differences between treatment groups in any of the other biochemical markers of bone turnover (PINP, CTx, or osteocalcin) at 26 weeks or 13 weeks (table 2). There were small statistically significant, but not clinically relevant, differences in lumbar spine BMD T-score at 26 weeks between the 50 μg group and placebo, and between the 50 μg group and 200 μg group. Total hip BMD did not differ between treatment groups at 26 weeks (appendix p 4).

There was a statistically significant but small difference in short physical performance battery score in the 50 μg group compared with placebo at 26 weeks, but no difference between the 200 μg group and placebo. Grip strength did not differ between treatment groups (table 4).

**Table 4:** Physical functions tests at baseline, 13 weeks, and 26 weeks by treatment group

	**Placebo**		**Selenite 50 μg**		**Selenite 200 μg**		**Selenite 50 μg *vs* placebo**		**Selenite 200 μg *vs* placebo**		**Selenite 200 μg *vs* 50 μg**	
	n	Mean	n	Mean	n	Mean	Difference	p value	Difference	p value	Difference	p value
**Short physical performance battery**												
Baseline	37	10·2 (9·7 to 10·7)	39	10·3 (9·7 to 10·9)	39	10·0 (9·6 to 10·5)	..	..	..	..	..	..
13 weeks	37	10·8 (10·5 to 11·3)	39	10·3 (9·8 to 10·8)	39	10·4 (9·7 to 11·0)	..	..	..	..	..	..
26 weeks	36	10·9 (10·5 to 11·4)	38	10·4 (9·9 to 10·9)	39	10·3 (9·7 to 10·8)	−0·5 (−1·1 to −0·03)	0·037	−0·5 (−1·0 to 0·05)	0·074	0·1 (−0·4 to 0·6)	0·76
**Grip strength dominant hand**												
Baseline	37	18·6 (17·3 to 20·0)	39	19·8 (18·3 to 21·3)	39	19·5 (17·9 to 21·1)	..	..	..	..	..	..
13 weeks	37	18·6 (17·3 to 20·0)	39	19·4 (18·0 to 20·8)	39	19·2 (17·9 to 20·5)	..	..	..	..	..	..
26 weeks	36	18·1 (16·6 to 19·5)	36	18·9 (17·4 to 20·4)	39	18·4 (16·9 to 19·8)	−0·3 (−1·2 to 0·6)	0·49	−0·3 (−1·2 to 0·6)	0·50	0·01 (−0·9 to 0·9)	0·99
**Grip strength non-dominant hand**												
Baseline	37	16·8 (15·5 to 18·2)	38	18·1 (16·8 to 19·3)	38	17·6 (16·3 to 18·9)	..	..	..	..	..	..
13 weeks	37	17·1 (15·8 to 18·4)	37	17·2 (16·0 to 18·4)	38	17·3 (15·7 to 18·8)	..	..	..	..	..	..
26 weeks	36	16·1 (14·7 to 17·5)	36	17·0 (15·8 to 18·2)	38	16·7 (15·1 to 18·3)	−0·7 (−1·6 to 0·2)	0·13	−0·3 (−1·2 to 0·6)	0·59	0·4 (−0·5 to 1·3)	0·40

Data are n, mean (95% CI), difference in means (95% CI), or p value. Difference in means for treatment group from mixed effects model adjusting for baseline measurement and after treatment time. Interactions between treatment group and after treatment time were not statistically significant (p>0·050) so estimates are for overall after treatment differences.

Glutathione peroxidase activity and highly sensitive C-reactive protein did not differ between treatment groups at 13 or 26 weeks (appendix p 5). Most IL-6 measurements did not reach the limit of detection of 1·6 ng/L (74 of 110 at baseline, 71 of 110 at week 13, and 74 of 108 at week 26), so no further analysis was done on the IL-6 measurements.

Dietary intake of vitamin D, calcium, and selenium assessed by 7-day diet diary were similar in all three treatment groups at baseline. Dietary selenium intake decreased between baseline and 26 weeks in all three groups but did not differ between groups at 26 weeks (appendix p 6). The safety assessments for diabetes and thyroid function did not differ between treatment groups. There was a small difference in glycated haemoglobin with treatment between 200 μg and placebo but this was not a clinically significant difference (appendix p 7). Seven (6%) of 120 participants were withdrawn from treatment at week 13 due to abnormal thyroid-stimulating hormone concentrations (one in the 200 μg group, three in the 50 μg group, and three in the placebo group) and one due to abnormal blood glucose (in the 50 μg group). The number and severity of adverse events, and the systems affected by adverse events were similar across treatment groups (appendix p 8). There were three serious adverse events: a non-ST elevation myocardial infarction at week 18 (in the 50 μg group), a diagnosis of bowel cancer after routine population screening at week 2 (in the placebo group), and a pulmonary embolus due to metastatic bowel cancer at week 4 (in the 200 μg group). All severe adverse events were judged by the principal investigator as unrelated to trial medication.

Analyses were repeated in the per-protocol group for all efficacy and safety endpoints, and the results did not differ from those in the primary analysis population.

## Discussion

We did a well powered randomised, double-blind, placebo-controlled study of the effects of selenium supplementation on musculoskeletal health in postmenopausal women and found that supplemental selenite 200 μg or 50 μg daily increased serum selenium concentrations but had no effect on biochemical markers of bone turnover, BMD, or physical function. This result is key because, to our knowledge, our study is the first randomised controlled trial of selenium supplementation for musculoskeletal health.

Serum selenium in the 200 μg treatment group increased from 80 μg/L to 105 μg/L. Mortality data suggest that the optimum range for serum selenium is 120–150 μg/L. Although serum selenium did not reach this range, based on the cross-sectional correlation of serum selenium and NTx to creatinine ratio in our previous study,^5^ an increase of 30% in serum selenium should be enough to cause a change in bone markers. Biochemical markers of bone turnover are dynamic and respond to bone active agents within days. For example, bone markers are decreased by about 20% 2 weeks after starting calcium supplements.^31^ Selenium 200 μg daily has been shown to be effective in Graves' eye disease^23^ and cancer prevention studies;^21^ therefore there is good evidence that this dose is high enough to be biologically active in humans. Higher dose supplements might have an effect on bone; however, across the two doses that we have studied, there was no dose-response effect. Higher doses might also increase the risk of adverse effects.^24^ Other forms of selenium supplementation might have different effects, but the common pathway is likely to be increased selenium availability.

There was a small difference in lumbar spine BMD between the 50 μg group and placebo, but in the absence of any effect on bone turnover or any BMD effect in the 200 μg group, this is likely to be a spurious result.

There are epidemiological, observational, and preclinical data to suggest that higher selenium intake might have beneficial effects on musculoskeletal health. Higher selenium status is associated with higher BMD in men in the Netherlands.^13^ Higher dietary selenium intake is associated with lower hip fracture risk in older adults in the USA^14^ and higher BMD in middle-aged and older adults in Europe.^5^, ^13^ Lower serum selenium and dietary selenium are associated with lower muscle mass and poorer muscle function in older adults.^17^

The proposed mechanism of action of selenium to reduce reactive oxygen species via increased biosynthesis of selenoproteins, and so reduce the pro-resorptive drive to osteoclasts was plausible. However, despite increased serum selenoprotein P, we saw no effect on markers of bone resorption. It might be that selenium status is a marker for other factors acting on bone health, or that a single factor approach is ineffective and selenium is part of a more complex system.

The population in this study was generally representative of postmenopausal women in the UK in terms of body-mass index, BMD, vitamin D status, and calcium intake. Their dietary selenium intake at baseline was higher than expected for the UK, but at 26 weeks was more typical.

We only studied women, and it is possible that the effects of selenium on bone would be different in men. However, postmenopausal women have higher bone resorption than men, and we hypothesised that selenium could act particularly through one of the resorption pathways activated by oestrogen deficiency. Therefore, in the absence of any effect in women, we do not think an effect in men is likely. These were healthy osteopenic women, and it is possible that selenium supplementation could have greater effect in women with underlying inflammatory disorders driving higher bone turnover.

Our outcome measures are surrogates (BMD and bone turnover for fracture and physical function tests for falls), but they are well validated surrogates, and the absence of effect in our study suggests that further studies with fall and fracture endpoints are probably not justified in this patient group.

Other trials have shown benefit in cancer prevention; thus selenium might have benefits for human health. However, we conclude that sodium selenite supplementation at these doses is unlikely to have beneficial effects on musculoskeletal health in postmenopausal women.

## Data sharing

All data requests should be submitted to the corresponding author for consideration. Access to anonymised data might be granted following investigator review, and with agreement from the NIHR.

## Declaration of interests

JSW declares grants from the NIHR during the conduct of the study; and personal fees from Mereo and Sandoz, personal fees and non-financial support from Eli Lilly, grants from Alexion, and non-financial support from Consilient, outside the submitted work. RMJ declares grants from the NIHR during the conduct of the study. LS declares shares in selenOmed, outside the submitted work. TRH declares speaker fees from DSM, outside the submitted work. JCM declares grants from the NIHR during the conduct of the study. RE declares grants from Amgen, Alexion, and Roche; grants and personal fees from IDS; personal fees from GSK Nutrition, Mereo, Sandoz, AbbiVie, Samsung, Haoma Medica, Elsevier, CL Bio, the Foundation for the National Institutes of Health, Viking, the University of California San Francisco, Biocon, and Lyramid; and grants and personal fees from Nittobo, outside the submitted work. GRW declares no competing interests.
