# Supplementary material for: Effect of selenium supplementation on musculoskeletal health in older women: a randomised, double-blind, placebo-controlled trial
Source: Lancet Healthy Longev. 2021 Apr;2(4):e212–21. doi: 10.1016/S2666-7568(21)00051-9 (PMC8020713; doi:10.1016/S2666-7568(21)00051-9)
Supplement: Supplementary appendix [file mmc1.pdf]

THE LANCET
Healthy Longevity

Supplementary appendix

This appendix formed part of the original submission and has been peer reviewed. We post it as supplied by the authors.

Supplement to: Walsh JS, Jacques RM, Schomburg L, et al. Effect of selenium supplementation on musculoskeletal health in older women: a randomised, double-blind, placebo-controlled trial. *Lancet Healthy Longev* 2021; published online March 23. https://doi.org/10.1016/S2666-7568(21)00051-9.

Supplementary material

Trial flow chart

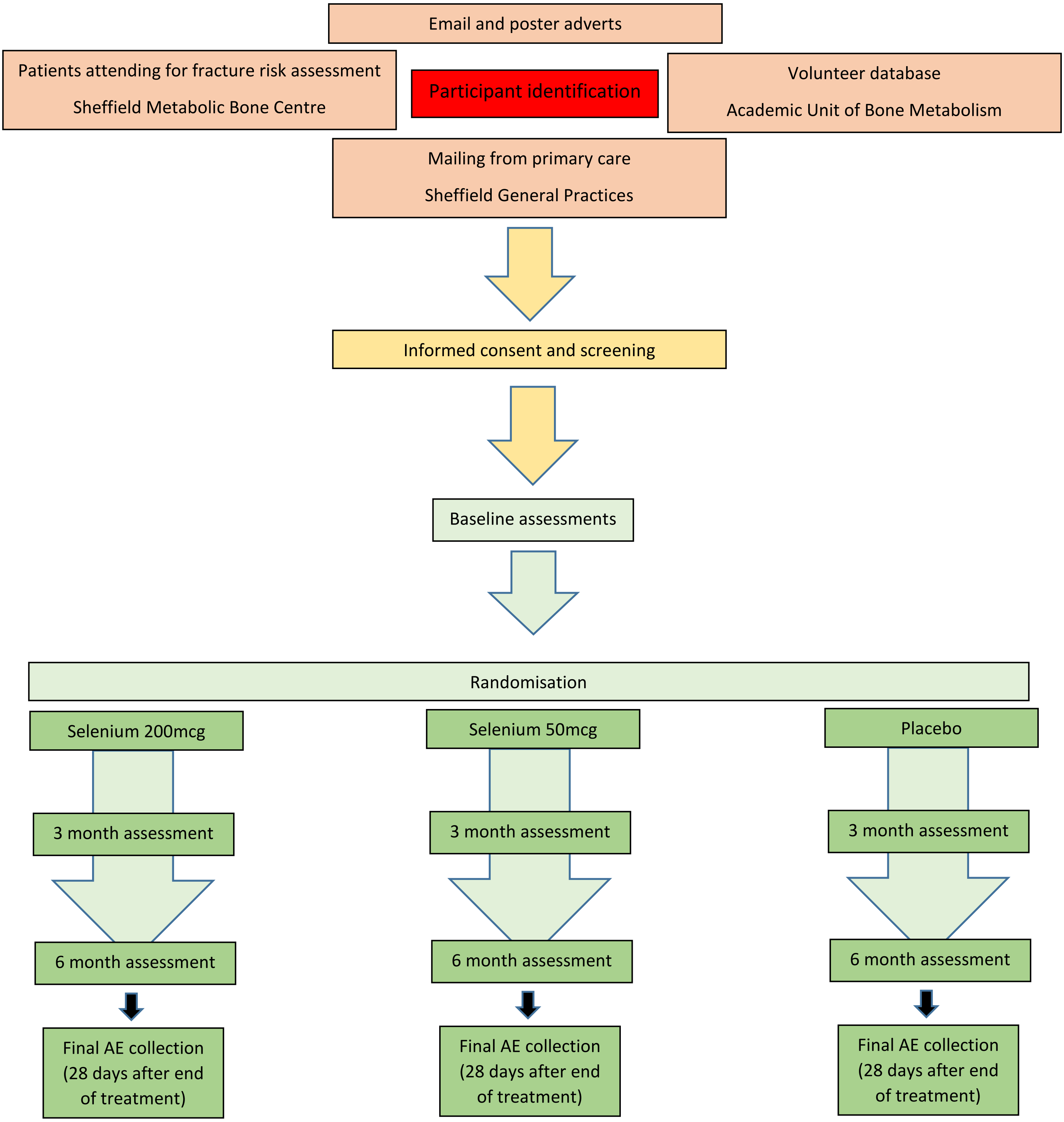

NTX/Cr by treatment group at 26 weeks and 13 weeks (per-protocol analysis)

	**Placebo**		**Selenase 50 mcg**		**Selenase 200 mcg**		**50 mcg – placebo**		**200 mcg – placebo**		**200 mcg – 50 mcg**	
	N	Mean[1] (95% CI)	N	Mean[1] (95% CI)	N	Mean[1] (95% CI)	Ratio[2] (95% CI)	P-Value	Ratio[2] (95% CI)	P-Value	Ratio[2] (95% CI)	P-Value
**NTX/Cr (nmolBCE/mmol) (Primary outcome)**												
Baseline	25	37.4 (31.8 to 44.0)	29	41.0 (36.2 to 46.5)	32	40.9 (36.0 to 46.6)	-	-	-	-	-	-
26 Weeks	25	42.4 (35.2 to 51.1)	29	44.3 (37.6 to 52.3)	32	40.6 (35.7 to 46.1)	0.98 (0.82 to 1.17)	0.80	0.90 (0.76 to 1.06)	0.21	0.92 (0.78 to 1.08)	0.29
**NTX/Cr (nmolBCE/mmol) (Secondary outcome)**												
Baseline	25	37.4 (31.8 to 44.0)	28	41.7 (36.8 to 47.2)	33	40.8 (36.0 to 46.3)	-	-	-	-	-	-
13 Weeks	25	41.2 (35.8 to 47.5)	28	43.4 (37.9 to 49.7)	33	42.3 (38.2 to 46.9)	0.98 (0.86 to 1.12)	0.77	0.97 (0.86 to 1.10)	0.62	0.99 (0.88 to 1.11)	0.85

[1] Geometric mean and 95% confidence interval

[2] Ratio of means for treatment group from ANCOVA model adjusting for baseline measurement. Outcome variable was log-transformed and the treatment group difference back transformed to give a ratio.

Serum Selenium at baseline, 13 weeks and 26 weeks by treatment group (ITT population)

Points are mean and bars are 95% CI

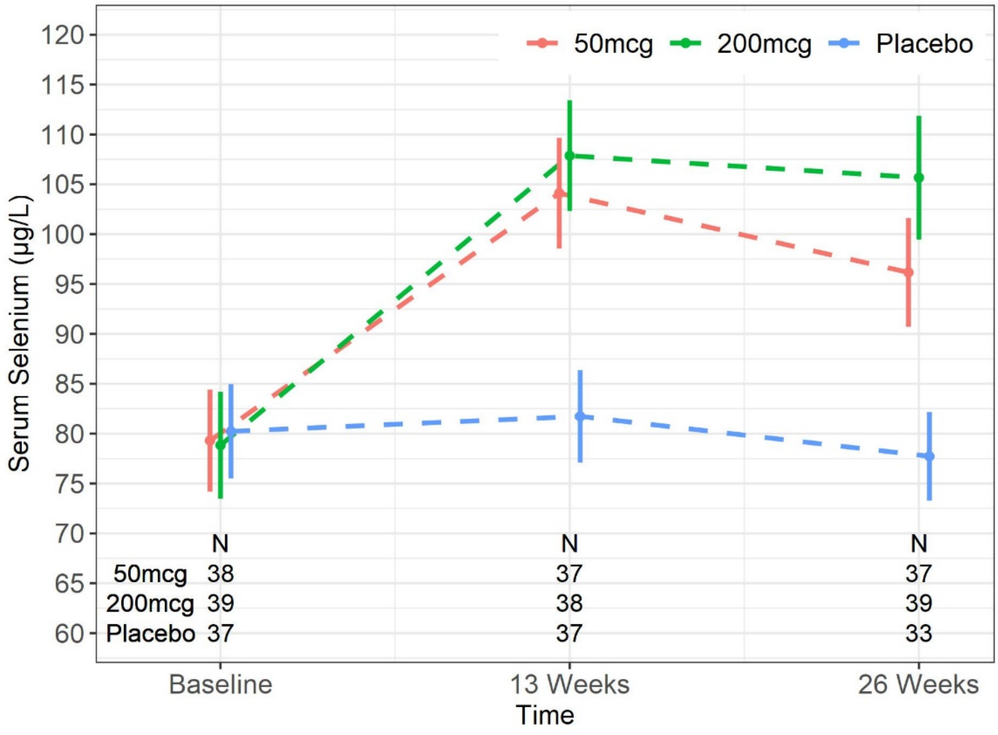

Serum Selenoprotein P at baseline, 13 weeks and 26 weeks by treatment group (ITT population)

Points are mean and bars are 95% CI)

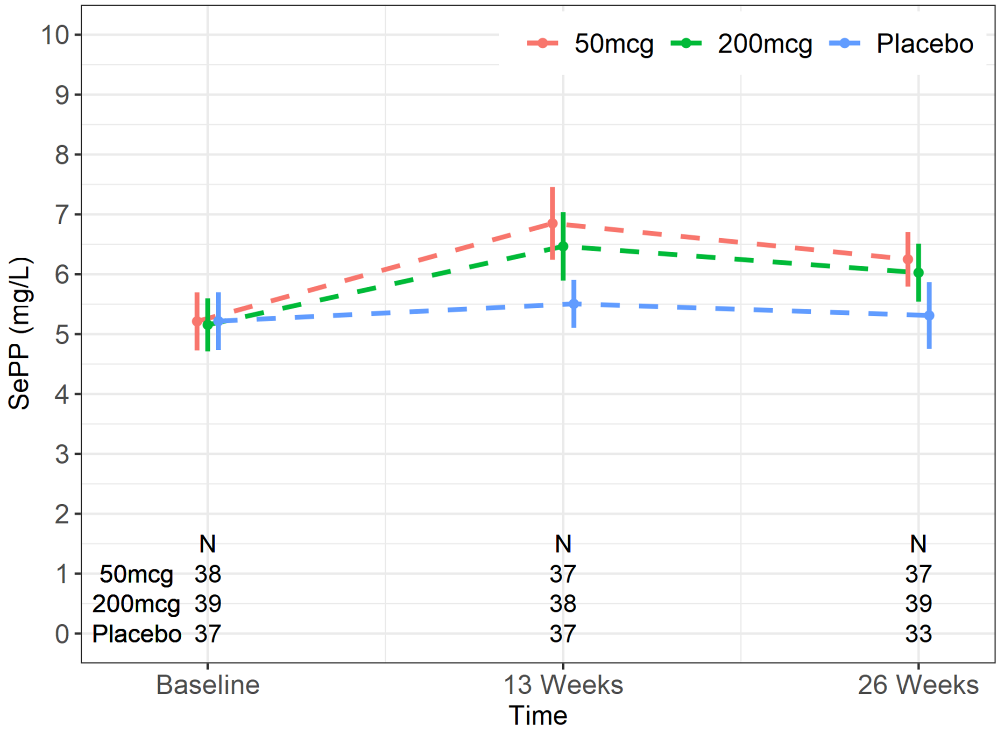

DXA bone mineral density at baseline and 26 weeks by treatment group (ITT analysis)

	**Placebo**		**Selenase 50 mcg**		**Selenase 200 mcg**		**50mcg – placebo**		**200 mcg – placebo**		**200 mcg – 50 mcg**	
	N	Mean[1] (95% CI)	N	Mean[1] (95% CI)	N	Mean[1] (95% CI)	Difference[2] (95% CI)	P-Value	Difference[2] (95% CI)	P-Value	Difference[2] (95% CI)	P-Value
**DXA T-Score total hip**												
Baseline	34	-1.28 (-1.51 to -1.04)	38	-1.23 (-1.45 to -1.00)	39	-0.91 (-1.11 to -0.70)						
26 Weeks	34	-1.22 (-1.45 to -0.99)	38	-1.18 (-1.40 to -0.96)	39	-0.89 (-1.09 to -0.69)	-0.003 (-0.12 to 0.11)	0.95	0.003 (-0.12 to 0.11)	0.96	0.01 (-0.11 to 0.12)	0.91
**DXA T-Score lumbar spine**												
Baseline	34	-1.70 (-2.01 to -1.39)	37	-1.87 (-2.18 to -1.57)	37	-1.84 (-2.04 to -1.63)						
26 Weeks	34	-1.78 (-2.08 to -1.47)	37	-1.78 (-2.08 to -1.48)	37	-1.89 (-2.11 to -1.67)	0.16 (0.03 to 0.29)	0.013	0.02 (-0.11 to 0.14)	0.80	-0.14 (-0.27 to -0.02)	0.021

[1] Arithmetic mean and 95% confidence interval
[2] Difference in means for treatment group from ANCOVA model adjusting for baseline measurement.

Anti-oxidant activity and inflammation by treatment group at 13 weeks and 26 weeks

	Placebo		Selenase 50 mcg		Selenase 200 mcg		50 mcg – placebo		200 mcg – placebo		200 mcg – 50 mcg	
	N	Mean[1] (95% CI)	N	Mean[1] (95% CI)	N	Mean[1] (95% CI)	Difference[2] (95% CI)	P-Value	Difference[2] (95% CI)	P-Value	Difference[2] (95% CI)	P-Value
**GPx activity (IU/l)** [1,2]												
Baseline	37	183.5 (152.4 to 214.5)	38	192.6 (156.4 to 228.8)	39	180.9 (155.1 to 206.8)	-	-	-	-	-	-
13 Weeks	37	184.4 (158.0 to 210.8)	37	204.2 (175.6 to 232.9)	38	180.7 (151.1 to 210.3)	-	-	-	-	-	-
26 Weeks	33	175.4 (147.5 to 203.2)	37	176.8 (149.2 to 204.3)	39	160.1 (132.4 to 187.8)	10.1 (-17.5 to 37.8)	0.47	-8.8 (-36.1 to 18.6)	0.53	-18.9 (-45.9 to 8.1)	0.17
**hsCRP (mg/l)** [3,4]												
Baseline	36	1.14 (0.80 to 1.63)	35	0.80 (0.55 to 1.15)	38	1.00 (0.65 to 1.41)	-	-	-	-	-	-
13 Weeks	36	1.12 (0.75 to 1.65)	35	0.77 (0.55 to 1.08)	38	1.04 (0.78 to 1.38)	-	-	-	-	-	-
26 Weeks	35	1.31 (0.88 to 1.94)	33	0.81 (0.58 to 1.13)	37	1.09 (0.74 to 1.60)	0.84 (0.63 to 1.15)	0.28	0.99 (0.74 to 1.32)	0.93	1.17 (0.87 to 1.57)	0.31

[1] Arithmetic mean and 95% confidence interval

[2] Difference in means for treatment group from mixed effects model adjusting for baseline measurement

[3] Geometric mean and 95% confidence interval

[4] Ratio of means for treatment group from mixed effects model adjusting for baseline measurement. Outcome variable was log-transformed and the treatment group difference back transformed to give a ratio.

Dietary intake of vitamin D, selenium and calcium by treatment group at baseline and 26 weeks

	Placebo		Selenase 50 mcg		Selenase 200 mcg		50 mcg – placebo		200 mcg – placebo		200 mcg – 50 mcg	
	N	Mean[1] (95% CI)	N	Mean[1] (95% CI)	N	Mean[1] (95% CI)	Ratio[2] (95% CI)	P-Value	Ratio[2] (95% CI)	P-Value	Ratio[2] (95% CI)	P-Value
**Dietary vitamin D (mcg/day)**												
Baseline	34	2.1 (1.5 to 2.8)	35	2.5 (2.0 to 3.0)	39	2.2 (1.8 to 2.8)	-	-	-	-	-	-
26 Weeks	34	1.9 (1.4 to 2.6)	35	2.2 (1.8 to 2.8)	39	2.0 (1.8 to 2.8)	1.06 (0.78 to 1.44)	0.73	1.02 (0.75 to 1.37)	0.92	0.96 (0.71 to 1.30)	0.80
**Dietary calcium (mg/day)**												
Baseline	34	769.9 (685.2 to 865.0)	35	850.7 (774.7 to 934.1)	39	795.5 (711.3 to 889.6)	-	-	-	-	-	-
26 Weeks	34	740.3 (664.6 to 824.7)	35	829.0 (736.9 to 932.5)	39	688.9 (596.3 to 795.9)	1.03 (0.91 to 1.18)	0.63	0.91 (0.78 to 1.03)	0.12	0.88 (0.78 to 1.00)	0.04
**Dietary selenium (mcg/day)**												
Baseline	34	78.7 (73.8 to 83.9)	34	79.6 (74.5 to 85.0)	39	76.9 (71.2 to 83.0)	-	-	-	-	-	-
26 Weeks	34	40.6 (35.4 to 46.5)	34	39.4 (34.3 to 45.1)	39	35.5 (31.1 to 40.7)	0.96 (0.81 to 1.16)	0.68	0.89 (0.75 to 1.06)	0.19	0.92 (0.77 to 1.10)	0.37

[1] Geometric mean and 95% confidence interval

[2] Ratio of means for treatment group from mixed effects model adjusting for baseline measurement. Outcome variable was log-transformed and the treatment group difference back transformed to give a ratio

Diabetes and thyroid function by treatment group at 13 weeks and 26 weeks

	Placebo		Selenase 50 mcg		Selenase 200 mcg		50 mcg – placebo		200 mcg – placebo		200 mcg – 50 mcg	
	N	Mean (95% CI)	N	Mean (95% CI)	N	Mean (95% CI)	Difference (95% CI)	P-Value	Difference (95% CI)	P-Value	Difference (95% CI)	P-Value
**Fasting blood glucose (mmol/l)** [1,2]												
Baseline	37	5.0 (4.8 to 5.2)	39	5.2 (4.9 to 5.5)	38	5.1 (4.9 to 5.3)	-	-	-	-	-	-
13 Weeks	37	4.9 (4.6 to 5.1)	37	5.1 (4.9 to 5.3)	37	4.9 (4.7 to 5.1)	0.2 (-0.1 to 0.4)	0.15	0.04 (-0.2 to 0.3)	0.71	-0.1 (-0.4 to 0.1)	0.28
26 Weeks	36	4.9 (4.7 to 5.1)	38	4.9 (4.9 to 5.1)	38	4.9 (4.8 to 5.1)						
**Insulin (pmol/l)** [3,4]												
Baseline	33	46.3 (37.4 to 57.4)	33	52.7 (41.8 to 66.6)	36	51.6 (41.8 to 63.7)	-	-	-	-	-	-
13 Weeks	32	46.0 (36.5 to 57.9)	31	54.0 (43.2 to 67.6)	35	55.5 (43.3 to 71.2)	0.99 (0.82 to 1.21)	0.94	1.05 (0.87 to 1.27)	0.62	1.06 (0.87 to 1.28)	0.58
26 Weeks	33	46.8 (38.3 to 57.2)	32	46.4 (37.7 to 57.1)	35	47.3 (38.8 to 57.6)						
**HbA1c (mmol/mol)** [1,2]												
Baseline	37	35.6 (34.8 to 36.4	39	36.0 (35.2 to 36.8)	39	36.2 (35.4 to 36.9)	-	-	-	-	-	-
13 Weeks	36	36.9 (34.9 to 38.9)	37	36.6 (35.8 to 37.3)	38	35.7 (34.8 to 36.6)	-0.3 (-1.3 to 0.6)	0.48	-1.0 (-2.0 to -0.1)	0.028	-0.7 (-1.6 to 0.2)	0.13
26 Weeks	36	36.3 (35.5 to 37.0)	38	36.6 (35.9 to 37.4)	37	36.1 (35.2 to 37.0)						
**TSH (mIU/l)** [3,4]												
Baseline	37	1.74 (1.51 to 2.00)	39	1.80 (1.55 to 2.11)	39	1.74 (1.48 to 2.04)	-	-	-	-	-	-
13 Weeks	36	2.19 (1.86 to 2.58)	37	2.41 (2.05 to 2.82)	36	2.31 (1.91 to 2.80)	0.96 (0.84 to 1.10)	0.57	0.97 (0.85 to 1.10)	0.68	1.01 (0.88 to 1.16)	0.87
26 Weeks	36	2.39 (2.04 to 2.81)	39	2.19 (1.87 to 2.57)	39	2.22 (1.84 to 2.69)						

[1] Arithmetic mean and 95% confidence interval

[2] Difference in means for treatment group from mixed effects model adjusting for baseline measurement and post treatment time.

[3] Geometric mean and 95% confidence interval

[4] Ratio of means for treatment group from mixed effects model adjusting for baseline measurement and post treatment time. Outcome variable was log-transformed and the treatment group difference back transformed to give a ratio.

Interactions between treatment group and post treatment time were not statistically significant (P>0.050) so estimates are for overall post treatment difference.

Adverse events by MedDRA system

	Placebo (N= 37)		Selenase 50mcg (N = 39)		Selenase 200mcg (N=39)		All (N=115)	
	Events N	Participants N (%)	Events N	Participants N (%)	Events N	Participants N (%)	Events N	Participants N (%)
**All**	34	23 (62.2)	34	27 (69.2)	27	17 (43.6%)	95	67 (58.3)
Infection and Infestation	10	9 (24.3)	8	8 (20.5)	10	7 (17.9)	28	24 (20.9)
Gastrointestinal	5	5 (13.5)	5	4 (10.3)	4	4 (10.3)	14	13 (11.3)
Musculoskeletal and Connective	3	3 (8.1)	6	5 (12.8)	3	3 (7.7)	12	11 (9.6)
Injury, Poisoning and Procedural Complications	7	6 (16.2)	1	1 (2.6)	1	1 (2.6)	9	8 (7.0)
Respiratory	3	2 (5.4)	2	2 (5.1)	2	1 (2.6)	7	5 (4.3)
Skin and Subcutaneous	1	1 (2.7)	3	3 (7.7)	2	2 (5.1)	6	6 (5.2)
Renal and Urinary	1	1 (2.7)	2	2 (5.1)	2	2 (5.1)	5	5 (4.3)
Neurological	1	1 (2.7)	2	2 (5.1)	1	1 (2.6)	4	4 (3.5)
Surgical and Medical Procedures	1	1 (2.7)	2	2 (5.1)	0	0 (0.0)	3	3 (2.6)
Vascular	0	0 (0.0)	3	2 (5.1)	0	0 (0.0)	3	2 (1.7)
Eye	1	1 (2.7)	0	0 (0.0)	0	0 (0.0)	1	1 (0.9)
General	0	0 (0.0)	0	0 (0.0)	1	1 (2.6)	1	1 (0.9)
Neoplasms	1	1 (2.7)	0	0 (0.0)	1	1 (2.7)	2	2 (1.8)
Psychiatric	0	0 (0.0)	0	0 (0.0)	1	1 (2.6)	1	1 (0.9)

The Effect of Selenium Supplementation on Musculoskeletal Health in Older Women

Double-blind, randomised, placebo-controlled trial

Acronym: SeMS

Protocol v1.1 16Feb 2017

**SPONSOR**	**Sheffield Teaching Hospitals NHS Foundation Trust**
**SPONSOR'S REFERENCE**	**STH19102**
**CHIEF INVESTIGATOR**	**Dr Jennifer Walsh**
**IRAS**	**200308**
**EUDRACT**	**2016-002964-15**
**Clinicaltrials.gov**	**NCT02832648**
**REC**	**16/YH/0393**
**FUNDER**	**NIHR EME 14-200-20**
**KEY WORDS**	**selenium, osteoporosis, bone turnover, muscle**

Contents

SIGNATURE PAGE 12

KEY TRIAL CONTACTSs 13

TRIAL SUMMARY 15

TRIAL FLOW CHART 1

LIST OF ABBREVIATIONS 17

1. RESEARCH QUESTION 19

2. LAY SUMMARY 19

3. BACKGROUND 19

5. HYPOTHESIS AND OBJECTIVES 21

6. RESEARCH DESIGN SUMMARY 21

7. STUDY POPULATION 22

7.1 Inclusion criteria 22

7.2 Exclusions 22

7.3 Bone density definitions 22

7.4 Generalisability 23

8. PLANNED INTERVENTIONS 23

9. OUTCOME MEASURES 24

10. ASSESSMENT AND FOLLOW-UP 24

10.1 Assessment of efficacy/effectiveness 24

10.2 Assessment of safety 25

10.3 Timing of assessments 25

11. TRIAL MEDICATION 26

12. RISKS AND BENEFITS 29

13. STATISTICS 30

13.1 Power calculation 30

13.2 Analysis plan 30

14. STUDY SETTING 31

15. ETHICAL CONSIDERATIONS 31

16. RECRUITMENT 32

16.1 Participant identification 32

16.2 Screening requirements 33

16.3 Consent 33

17. RANDOMISATION 34

18. BLINDING 34

19. PHARMACOVIGILANCE 35

19.1 Reporting Period 35

19.2 Definitions ... 35
19.3 Causality ... 35
19.4 Responsibilities ... 36
20. WITHDRAWAL ... 36
21. SAMPLE STORAGE AND ANALYSIS ... 36
22. DATA PROTECTION ... 37
23. GOVERNANCE AND MANAGEMENT ... 37
24. TIMELINES ... 38
25. PATIENT AND PUBLIC INVOLVEMENT ... 39
REFERENCES ... 40
APPENDIX 1 – Physical function tests ... 42
APPENDIX 2 – Schedule of procedures ... 45

SIGNATURE PAGE

The undersigned confirm that the following protocol has been agreed and accepted and that the Chief Investigator agrees to conduct the trial in compliance with the approved protocol and will adhere to the principles outlined in the Medicines for Human Use (Clinical Trials) Regulations 2004 (SI 2004/1031), amended regulations (SI 2006/1928) and any subsequent amendments of the clinical trial regulations, GCP guidelines, the Sponsor's SOPs, and other regulatory requirements as amended.

I agree to ensure that the confidential information contained in this document will not be used for any other purpose other than the evaluation or conduct of the clinical investigation without the prior written consent of the Sponsor

I also confirm that I will make the findings of the study publically available through publication or other dissemination tools without any unnecessary delay and that an honest accurate and transparent account of the study will be given; and that any discrepancies from the study as planned in this protocol will be explained.

For and on behalf of the Study Sponsor:

Signature: .... Date: ....../....../......

Name (please print):

....

Position:

....

Chief Investigator:

Signature: .... Date: ....../....../......

Name: (please print):

....

Statistician:

Signature: ....

Name: (please print):

....

KEY TRIAL CONTACTS

Chief Investigator	Dr Jennifer Walsh<br>Senior Clinical Lecturer<br>Mellanby Centre for Bone Research<br>University of Sheffield<br><br>Metabolic Bone Centre<br>Sorby Wing<br>Northern General Hospital<br>Herries Road<br>Sheffield<br>S5 7AU<br><br>0114 2714705<br>j.walsh@sheffield.ac.uk
Trial Co-ordinator	Selina Bratherton<br>Metabolic Bone Centre<br>Sorby Wing<br>Northern General Hospital<br>Herries Road<br>Sheffield<br>S5 7AU<br><br>0114 3052005<br>s.bratherton@sheffield.ac.uk
Sponsor	Sheffield Teaching Hospitals NHS Foundation Trust<br><br>Clinical Research Office<br>D floor<br>Royal Hallamshire Hospital<br>Glossop Road<br>Sheffield<br>S10 2JF<br><br>0114 2265945
Funder(s)	National Institute for Health Research (EME)<br><br>Evaluation, Trials and Studies Coordinating Centre<br>University of Southampton<br>Alpha House, Enterprise Road<br>Southampton<br>SO16 7NS<br><br>02380 594309<br>m.r.shergill@soton.ac.uk

Key Protocol Contributors	Professor Richard Eastell<br>Mellanby Centre for Bone Research<br>University of Sheffield<br><br>Professor John Mathers<br>Institute for Ageing and Health<br>University of Newcastle<br><br>Dr Tom Hill<br>School of Agriculture, Food and Rural Development<br>University of Newcastle<br><br>Professor Graham Williams<br>Department of Medicine<br>Imperial College, London
Statistician	Dr Richard Jacques<br>ScHARR<br>University of Sheffield<br><br>0114 2220813<br>r.jacques@sheffield.ac.uk
Trials pharmacist	Kim Ryalls<br>Sheffield Teaching Hospitals NHS Foundation Trust<br><br>0114 2715456<br>kim.ryalls@sth.nhs.uk
Research Ethics Committee	Yorkshire and the Humber – Sheffield

TRIAL SUMMARY

Trial Title	The Effect of Selenium Supplementation on Musculoskeletal Health in Older Women	
Short Title	SeMS	
Trial Design	Double-blind, randomised, placebo controlled	
Trial Participants	Postmenopausal women with osteopenia or osteoporosis	
Planned Sample Size	120 (max 165)	
Treatment duration	6 months	
Planned Trial Period	26 months	
	Objectives	Outcome Measures
Primary	Decrease in bone resorption	Urine NTX
Secondary	Increased selenium<br>Decrease in bone turnover<br>Increase in physical functioning<br>Increased antioxidant activity<br>Decreased inflammatory markers	Serum Se, SePP<br>Serum CTX, PINP, osteocalcin<br>SPPB score, grip strength<br>Glutathione peroxidase, hydroperoxidase<br>IL-6, hsCRP
Investigational Medicinal Product(s)	Selenase (Biosyn, Germany)<br>Sodium selenite pentahydrate	
Dose<br>Route of Administration	200mcg daily, 50mcg daily, placebo<br>Oral	

TRIAL FLOW CHART

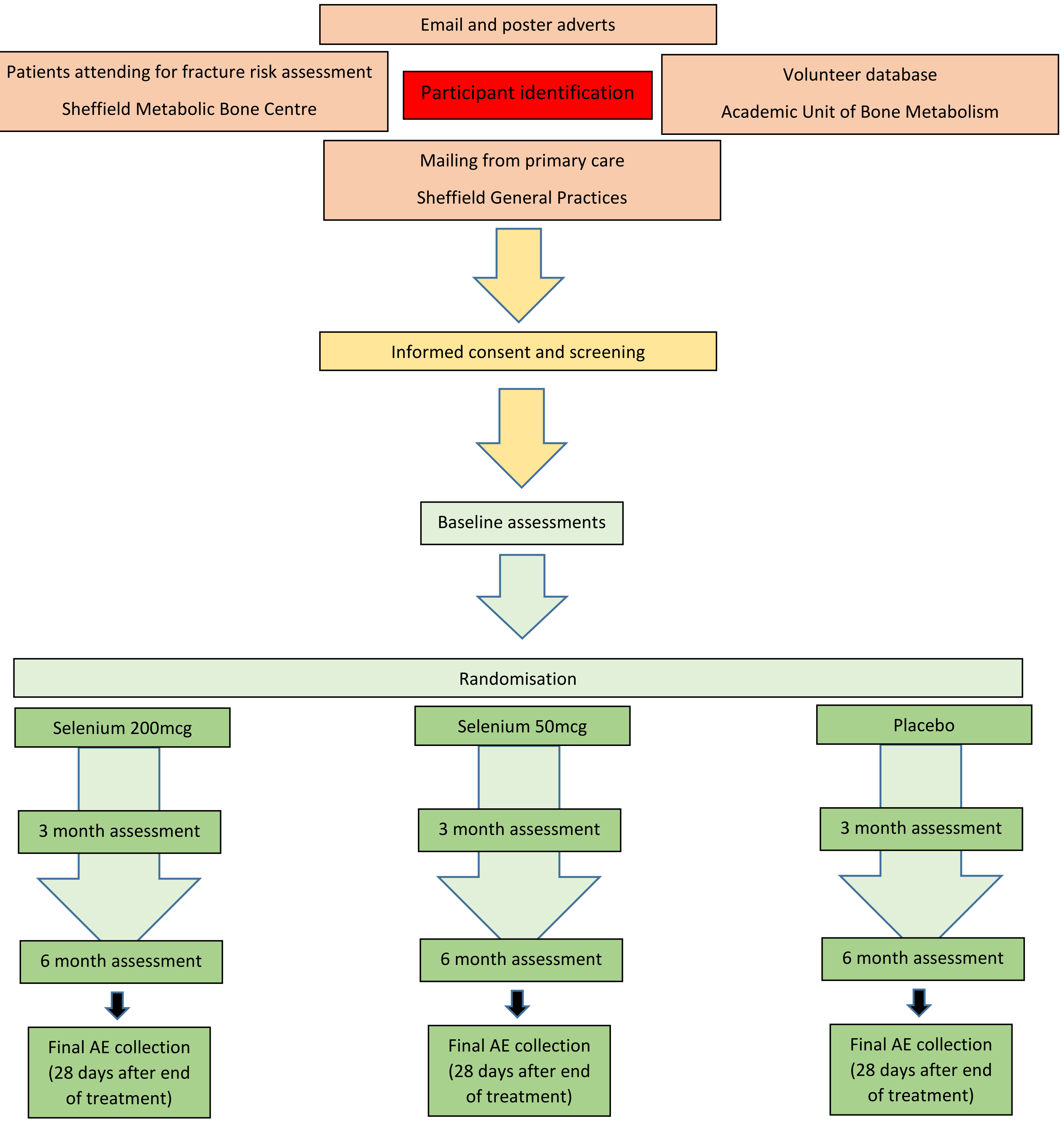

LIST OF ABBREVIATIONS

AE	Adverse Event
AR	Adverse Reaction
AUBM	Academic Unit of Bone Metabolism, Sheffield
BMD	Bone Mineral Density
CRF	Case Report Form
CRF	Clinical Research Facility
CTA	Clinical Trial Authorisation
CTIMP	Clinical Trial of Investigational Medicinal Product
CTX	C-terminal cross-linking telopeptide of type I collagen
DMEC	Data Monitoring Committee
DSUR	Development Safety Update Report
DXA	Dual Energy X-ray Absorptiometry
EudraCT	European Clinical Trials Database
GCP	Good Clinical Practice
GMP	Good Manufacturing Practice
HbA1c	Gylcated haemoglobin
HRA	Health Research Authority
HRT	Hormone Replacement Therapy
hsCRP	highly sensitive C-Reactive Protein
HTA	Human Tissue Act
IB	Investigator Brochure
ICH	International Conference on Harmonisation of technical requirements for registration of pharmaceuticals for human use.
IL-6	Interleukin-6
IMP	Investigational Medicinal Product
IMPD	Investigational Medicinal Product Dossier
ISF	Investigator Site File
ITT	Intention to Treat
MHRA	Medicines and Healthcare products Regulatory Agency
NHS R&D	National Health Service Research & Development
NIHR	National Institute for Health Research
NTX	N-terminal cross-linking telopeptide of type I collagen
OC	Osteocalcin

PINP	Procollagen type I N propeptide
PI	Principal Investigator
PIC	Participant Identification Centre
PIS	Participant Information Sheet
PPI	Patient and Public Involvement
QA	Quality Assurance
QC	Quality Control
RCT	Randomised Control Trial
REC	Research Ethics Committee
RSI	Reference Safety Information
SAE	Serious Adverse Event
SAR	Serious Adverse Reaction
SePP	Selenoprotein P
SERM	Selective Estrogen Receptor Modulator
SOP	Standard Operating Procedure
SmPC	Summary of Product Characteristics
SPPB	Short Physical Performance Battery
STH	Sheffield Teaching Hospitals
SUSAR	Suspected Unexpected Serious Adverse Reaction
TMG	Trial Management Group
TSH	Thyroid Stimulating Hormone
TSC	Trial Steering Committee

1. RESEARCH QUESTION
Does selenium supplementation decrease bone turnover or improve muscle function in postmenopausal women with osteopenia or osteoporosis?

2. LAY SUMMARY
This research aims to determine whether selenium supplements improve bone and muscle health in older women at risk of osteoporosis (low bone density or weak bones) and fracture (broken bones).

Osteoporosis is a major public health problem. One in two women and one in five men over age 50 will have a fracture. Fractures cause pain, disability and reduce life-expectancy. Women with below-average bone density around the time of the menopause might have previously taken hormone replacement (HRT) to prevent osteoporosis, but HRT is much less used now due to side effects. Therefore there is a need for safe, effective and inexpensive preventative interventions for women at risk of osteoporosis.

Selenium is a chemical nutrient present in several human proteins, including anti-oxidants. Anti-oxidants may protect against ageing of tissues, including bone, by mopping up damaging reactive oxygen molecules (sometimes called 'free radicals'). Selenium is present in soil, and so is obtained from many foods. However, soil selenium levels are low in Europe, and dietary intake in the UK is below recommended levels.

We previously found that women with higher blood selenium levels have stronger bones, but this doesn't prove that giving selenium will improve bone strength.

We propose a randomised controlled trial to compare selenium supplements with a placebo (dummy treatment) in women with below-average bone density. We will give selenium (at two different doses) or placebo to 120 women for six months and use blood and urine tests and bone density scans to see if giving selenium does have any effect on bone. We will also do muscle function tests and measurements of free radical molecules.

3. BACKGROUND
One in two women and one in five men over the age of fifty will have a fragility fracture. Fractures lead to pain, disability, loss of independence and increased mortality. This is a huge health burden for affected individuals, the NHS and social care which is increasing as the population ages.

About 30% of women aged over 65 are osteopenic (below-average bone density), and at risk of developing osteoporosis and fractures. More than 50% of all fractures in postmenopausal women occur in women with osteopenia. These women are not eligible for osteoporosis treatment at present because of the individual risk/benefit ratio and economic factors. Previously, these women could have been offered hormone replacement therapy (HRT) for bone protection, but adverse effects of HRT have limited its use.

Therefore there is a need for an effective, safe, well-tolerated, inexpensive and widely applicable preventative option for osteopenic women.

Selenium is a chemical element present in several human proteins. Twenty-five human selenoproteins have been identified (1). Known functions of selenoproteins include thyroid hormone synthesis (iodothyronine deiodinases) and antioxidants (thioredoxin reductases and glutathione peroxidases) (2, 3). Selenoproteins are anti-inflammatory and antioxidant; they reduce interleukin-6 and reactive oxygen species both of which are potent stimuli for bone resorption (4-6).

Selenium is obtained from the diet and it is rich in seafood, meat and cereals. The main determinant of food selenium content is soil selenium content. The recommended adequate intake for adults aged over 50 in the UK is 75ug/d for men and 60ug/d for women (7), but in the UK, the mean intake is only 40mcg/day (8). Selenium intakes have been declining in the UK in the past few decades, and are generally low in Europe compared with the USA. The main reason for the decreasing intake in the UK is a change in the source of flour for bread-making from North

America (which contains higher selenium) to Europe due to economic and consumer demands. More recently, the levels of selenium in UK soils have declined as a result of changes in fertiliser practice (e.g. replacing single superphosphate with triple superphosphate) and reduced industrial emissions.

We recently reported that in 1144 older women from the UK, France and Germany, higher serum selenium or selenoprotein P was associated with higher bone mineral density of the lumbar spine and total hip, and lower biochemical markers of bone turnover (9). High bone turnover is the principal mechanism of osteoporotic bone loss. We also noted associations of selenium levels with balance, grip strength and pulse rate. Others have reported an association of selenium with muscle strength (10), but we were the first to explore bone turnover or bone density. Selenium status was inversely related to thyroid hormone status (selenium is required for thyroid hormone synthesis), but the associations of selenium with bone measures were independent of thyroid hormones.

It is plausible that selenium would affect bone metabolism. Selenoproteins are found in osteoblasts and osteoclasts (11). Selenoproteins are anti-inflammatory and reduce interleukin-6, a potent stimulus for bone resorption (5). Selenoproteins are anti-oxidant and reduce reactive oxygen species; these also stimulate bone resorption via increased RANK-L signalling (11). An increase in reactive oxygen species has been proposed as a key mechanism by which sex hormone deficiency causes age-related bone loss through the same RANK pathway (12). Therefore is possible that selenium could directly antagonise the cellular mechanism of postmenopausal osteoporosis.

There is experimental animal evidence to support the hypothesis that selenium has a role in bone and reduces bone turnover. Selenium deficient mice have poorer bone microarchitecture, higher bone resorption markers and higher inflammatory markers than selenium supplemented mice (13). Selenium deficient rats have poor bone microarchitecture and abnormal skeletal growth (14, 15).

There may also be an epidemiological association between selenium deficiency and bone health. In a study of US hip fracture risk in women over age 65 years, the counties with highest rates were in a belt across the south of the USA and the lowest rates were in the north (16). In contrast, a current map of soil selenium content in the USA shows the highest selenium content in the north of the USA and the lowest in a belt across the south of the USA (http://mrdata.usgs.gov/geochem/doc/averages/se/usa.html).

Endemic selenium deficiency in humans has been associated with the osteoarthropathy Kashin-Beck disease (17).

Several other age-related disorders are linked to inadequate selenium status, including poor cognitive function (4) and selenium may be an independent predictor of mortality among older community-dwelling adults (18).

The possible adverse effects of selenium supplementation are thyroid dysfunction (because some selenoproteins are involved in thyroid hormone synthesis) and increased risk of type 2 diabetes mellitus (although in a previous study at the same dose and duration of treatment as this protocol there was no increase in diabetes (19)).

4. RATIONALE FOR CURRENT STUDY

If selenium supplementation were effective in reducing bone turnover and increasing BMD, this would offer an affordable, low risk prevention strategy that could be attractive to patients because it is a nutritional supplement, rather than a ‘medicine’.

This study will give clear evidence of efficacy, and if positive, will identify the likely effective dose.

If this study is positive, we will immediately apply for funding to take the best dose (the lowest dose with a clinically significant clinical effect on bone turnover) into a longer study with bone density as the primary endpoint.

5. HYPOTHESIS AND OBJECTIVES

Hypothesis: Selenium supplementation decreases bone turnover in postmenopausal women with low bone density by reducing pro-resorptive reactive oxygen species.

Purpose: to determine if selenium supplementation is beneficial for osteoporosis prevention and muscle function in postmenopausal women.

Objectives: to determine if six months selenium supplementation in postmenopausal women with osteopenia or osteoporosis

- decreases bone turnover
- increases bone density
- improves physical function score and grip strength
- is safe (particularly for thyroid function and diabetes)
- is well-tolerated
- decreases markers of oxidative stress and inflammation

All of these objectives will be answered at the completion of the study and analysis, with a total project duration of 26 months.

6. RESEARCH DESIGN SUMMARY

This is a double-blind, randomised, placebo controlled trial.

We will evaluate two doses of selenium (50 and 200mcg daily) vs placebo over six months.

Participants are postmenopausal women with osteopenia or osteoporosis (T-score -1.0 to -3.0).

We will include participants with any baseline serum selenium concentration for generalisability, but the primary endpoint efficacy analysis will only include women with baseline serum selenium below 120mcg/l.

Primary endpoint: Urinary N-telopeptide of type I collagen (NTX/Cr).

Secondary endpoints: other bone turnover markers, BMD, muscle function, thyroid function, blood glucose, anti-oxidant activity, inflammatory markers

We will make the primary analysis based on women with baseline serum selenium below 120mcg/l. To ensure adequate power for this analysis we will plan to recruit 120 women (we expect that 100 of the 120 will have serum selenium below 120 mcg/l based on our previous cross-sectional study). We will review the baseline serum selenium results when 40 women have been recruited to confirm the expected distribution of baseline levels, and we will adjust the total recruitment number accordingly to ensure at least 99 women with serum selenium below 120mcg/l have been randomised for the final primary endpoint intention-to-treat analysis.

Participants will be randomised according to a schedule produced by Sharp Clinical Services. Randomisation will be carried out independently of the study investigators. The blind will only be broken if judged by the PI as clinically necessary for the wellbeing of a participant.

If at the interim analysis, less than 60% of women recruited have serum selenium below 120 mcg/l the study recruitment strategy will be reviewed and discussed with the funder to determine if the trial should proceed.

The study may be stopped early if the investigators, sponsor or DMEC identify a safety concern.

Trial management will be done by the Academic Unit of Bone Metabolism CTIMP group (who meet monthly), and progress reported to the AUBM management group and Lay Panel. We will establish a TSC and DMEC.

7. STUDY POPULATION

Participants will be recruited from an existing volunteer database, patients attending the Sheffield Metabolic Bone Centre, mailing from GP surgeries and email and poster adverts.

7.1 Inclusion criteria

- Women
- Aged over 55, and at least 5 years since last menstrual period.
- Osteopenia or osteoporosis (DXA BMD lowest T-score between -1.0 and -3.0 at lumbar spine or total hip), who do not require pharmacological treatment for fracture prevention
- Willing and able to give informed consent

7.2 Exclusions

- Diabetes mellitus
- Thyroid dysfunction (history of hyper- or hypothyroidism, or TSH outside the STH reference range)
- any conditions known to affect bone metabolism
    - inflammatory disease (inflammatory arthritis, inflammatory bowel disease)
    - parathyroid disease
    - malabsorption (coeliac disease, pancreatic exocrine insufficiency)
    - alcohol intake >21 units per week
    - prolonged immobility (in bed or using a wheelchair for more than three months within the last five years)
    - fracture in the last year
    - major orthopaedic surgery in the last year
- Taken selenium supplements in the last 60 days (women who are taking selenium supplements but want to participate in the trial will be offered the option to stop their supplement and have a 60 day washout before screening).
- Previous adverse reaction to selenium or any of the IMP or placebo excipients
- Current or recent medications known to affect bone metabolism
    - Bisphosphonates (12m before)
    - Teriparatide (12m before)
    - Denosumab (12m before)
    - Oestrogen or any sex steroids (12m before)
    - Aromatase inhibitors (12m before)
    - Tamoxifen or raloxifene (12m before)
    - Active vitamin D metabolites; calcitriol, 1α-calcidol (6m before)
    - Anti-epileptics; phenytoin, carbamazepine, primidone, valproate (6m before). Low dose use for migraine or neuralgia is not an exclusion.
    - Oral, IM or IV glucocorticoids (6m before)
- Women taking calcium and vitamin D supplements will not be excluded as long as they have been taking the calcium and vitamin D for at least 60 days and are planning to continue throughout the trial.

7.3 Bone density definitions

Normal bone density is defined as T-score above -1.0.

Women with normal bone density are not eligible to enter the study.

Osteopenia is defined as T-score -1.0 to -2.5.

Most women with osteopenia do not receive treatment with bisphosphates or other medication because their fracture risk is not high enough to justify the cost and exposure to side-effects. These women will be eligible to enter the study.

Some women with osteopenia (T-score -1.0 to -2.5) do require pharmacological treatment because factors other than BMD indicate a high risk of further fracture (eg prevalent vertebral fracture). These women will not be eligible to enter the study.

Osteoporosis is defined as T-score below -2.5.

Treatment with bisphosphonates or other medication is advised for most women with osteoporosis. These women will not be eligible to enter the study.

Some women with osteoporosis do not require pharmacological treatment because factors other than BMD indicate that they are not yet at high risk of fracture (eg relatively young age with no previous fractures, no other risk factors). These women will be eligible to enter the study.

Some women with osteoporosis are recommended to receive treatment with bisphosphonates or other medication, but decide not to take treatment (eg because of concerns about side-effects). These women will be eligible to enter the study.

Patients recruited through the Metabolic Bone Centre will have already have had BMD measurement with fracture risk assessment and a treatment recommendation from a Metabolic Bone Centre doctor.

Potential participants (from GP mailing and adverts) who have not previously had BMD and fracture risk assessment will have BMD and fracture risk assessment at screening. The PI (who is also a Metabolic Bone Centre doctor) will make the assessment according the same criteria as patients attending the Metabolic Bone Centre. If the potential participant is at high risk of fracture and requires treatment they will be excluded from the study and the PI will write to the patient and their GP with treatment advice.

7.4 Generalisability

We will measure baseline serum selenium levels to explore the relationship between baseline selenium levels and response to supplementation, but we will not exclude participants based on their serum levels. By having an unselected group can we explore whether there is a subset that should be targeted or whether everyone benefits. If selenium supplementation proves to be an effective preventative measure for women with osteopenia, a requirement for baseline selenium measurement might impede widespread adoption, and potential implementation as a public health measure.

To ensure all participants are vitamin D replete, they will be given a single oral dose of 100,000IU colecalciferol at the screening visit (NIMP).

8. PLANNED INTERVENTIONS

The intervention is a medicinal product containing sodium selenite. We will study two doses: 50 and 200mcg daily. The preparation is sodium selenite tablets ‘Selenase’ 50mcg and 200mcg (Biosyn, Germany). The tablets will be overencapsulated and a matching placebo will be manufactured to maintain the blind.

The mean difference between selenium levels in a replete population (USA) and the UK is about 60mcg/L. The mean increase in serum selenium with 200mcg/day is 90 mcg/L (19). 200mcg/day is a clinically effective dose in Graves’ ophthalmopathy. Therefore 200mcg will be enough to achieve repletion in the majority of subjects and to determine whether selenium repletion has a clinically significant effect on bone turnover. If we see no effect at this dose we will be confident to accept the null hypothesis.

The 50mcg dose will assess whether a lower dose might be effective for musculoskeletal health – if its effect on bone turnover is similar to the 200mcg dose we could recommend the 50mcg dose for clinical use, at lower cost and lower risk of adverse effects.

This is a short, low risk study of a medication that we expect to be well tolerated. We are very experienced in recruiting and retaining patients in clinical trials. We expect compliance to be good and loss to follow-up to be low (less than 10%). We will make telephone contact with participants between study visits (every month) to maintain their engagement and check for adverse effects.

The required minimum sample size for the primary endpoint is 21 per group. We will recruit 33 per group into the primary analysis to allow for drop-out, group imbalance and secondary endpoint analysis.

9. OUTCOME MEASURES

Primary endpoint:

Between-group difference in urinary N-telopeptide of type I collagen (NTX) at six months

Bone turnover markers change much more rapidly than BMD, so we can determine quickly and cost-effectively if selenium is likely to have beneficial effects, and if it is well tolerated and safe in the short term.

We chose NTX because we know how change in NTX relates to fracture risk reduction with bisphosphonates (20): a 30% decrease in NTX is associated with a 40% reduction in spine fracture and 66% of the vertebral fracture risk reduction at three years is explained by change in NTX. Also, NTX was the marker mostly strongly related to selenium status in our observational study (9).

Secondary endpoints:

Change in serum selenium, selenoprotein P (SePP). Systematic review identified blood selenium and selenoprotein P as robust biomarkers of selenium status, over the range of deficiency to repletion (21).

Change in other bone turnover markers: PINP, osteocalcin, CTX

Change in bone mineral density: lumbar spine and total hip by DXA

Change in muscle function: short physical performance battery (SPPB), hand grip strength. SPPB score is a measure of lower limb strength and balance. It predicts falls, loss of function in activities of daily living, nursing home admission and mortality (22-24).

Change in anti-oxidant activity: glutathione peroxidase activity (a selenium-containing anti-oxidant which is increased in postmenopausal women with osteopenia (25)), hydroperoxides (an oxidative stress marker shown to be associated with bone resorption (26))

Change in inflammatory markers: the pro-resorptive inflammatory cytokine interleukin-6 and highly-sensitive C-reactive protein.

10. ASSESSMENT AND FOLLOW-UP

10.1 Assessment of efficacy/effectiveness

Efficacy measurements will be obtained at baseline, three months and six months.

Blood samples for biochemical measurements will be taken fasted. The approximate maximum blood sample volume at each visit will be 50ml. Urine samples will be obtained as triplicate samples from fasted second morning voids on three days before or including the study visit, and kept refrigerated until the study visit. Equal volume aliquots from the urine samples will be pooled into a single sample by the study team. Samples will processed according to a sample handling SOP.

Real-time baseline serum selenium will be measured in the Chemical Pathology laboratory, Sheffield Teaching Hospitals.

All other samples will be frozen at -80°C, and analysed in batches at the end of the study.

Urine NTX/Cr will be measured by automated assay (EC Vitros) in the laboratory of the Sheffield Academic Unit of Bone Metabolism or Chemical Pathology laboratory, Sheffield Teaching Hospitals.

Serum selenium at all time points will be measured by X-ray fluorescence spectroscopy (27), selenoprotein P will be measured by immunoassay (28) and glutathione peroxidase by enzyme analysis by Prof Lutz Schonberg, Institute for Experimental Endocrinology, Charité - University Medical School Berlin.

Other biochemical markers of bone turnover (serum CTX, PINP and osteocalcin) will be measured by automated assay in the laboratory of the Sheffield Academic Unit of Bone Metabolism.

HsCRP and IL-6 will be measured by the Sheffield Teaching Hospitals Clinical Immunology laboratory.

Hydroperoxidases will be measured using commercially available manual assay kits in the laboratory of the Sheffield Academic Unit of Bone Metabolism.

Grip strength will be assessed using a digital hand dynamometer (Saehan Corporation, Masan, Korea). Three measurements will be taken and the best value used for analysis. Short physical performance battery (SPPB) score will be calculated from a chair stand and narrow walk test (22). *See Appendix 1 – Physical function tests*.

Height and weight will be measured with an electric scale and stadiometer.

Pulse and blood pressure will be measured with an automated sphygmomanometer (Dynamap).

Bone mineral density will be assessed by DXA of the spine and hip (Hologic) at baseline and six months according to standard scanning protocols, by specialist DXA scan technicians in the Sheffield Clinical Research Facility.

Habitual dietary selenium and other nutrient intake (calcium, vitamin D, vitamin E and iodine) will be assessed with seven-day diet diaries. The purpose of the food diaries is to describe participants' habitual dietary intake of selenium and nutrients which influence bone turnover or interact with selenium during the study. The diaries will be analysed using DIETQ (Tinuviel Software, Warrington, UK) by a nutritionist with experience in clinical research.

DNA will be stored for later analysis. There is evidence of glutathione peroxidase genotype influence on selenium action (29). If selenium does reduce bone turnover we can study genotype interactions to better understand its mechanism of action.

10.2 Assessment of safety

Safety assessments for diabetes and thyroid function will be made at screening, baseline, three months and six months. The measurements will be made in real time by Sheffield Teaching Hospitals pathology laboratories. Any parameters outside the reference range will be assessed by the PI or a delegated study doctor for a decision on whether a participant can safely continue, and clinical advice will be given to the participant and GP as required.

Adverse events (including questioning for possible symptoms of selenium toxicity) will be collected from the time of consent, at monthly participant telephone contact throughout the treatment period and by telephone four weeks after the end of treatment.

10.3 Timing of assessments

Baseline and randomisation - within 28 days of screening visit

4W - baseline +28 days (+/-7 days)

8W - baseline +56 days (+/-7 days)

13W - baseline +91 days (window +73 to +94 days)

17W - baseline +119 days (+/-7 days)

21W - baseline +147 days (+/-7 days)

26W - baseline + 182 days (window +172 to +187 days)

30W - 26W + 28 days (+7 days)

See Appendix 2 – Schedule of procedures

11. TRIAL MEDICATION

11.1 Name and description of investigational medicinal product(s)

Selenase 50mcg and Selenase 200mcg (Biosyn, Germany)

Sodium selenite pentahydrate tablets, over-encapsulated.

11.2 Legal status of the drug

The trial is being carried out under a Clinical Trial Authorisation (CTA). The drug is therefore only to be used by the named investigators, for the patients specified in this protocol, and within the trial.

11.3 Summary of Product Characteristics (SmPC)

An IMPD will be submitted to the MHRA who will approve its use in this study. It will be provided by Sharp Clinical Services UK and contain the SmPC for Selenase (translated to English).

The SmPC will be used as the reference safety information (RSI) for assessment of adverse events and classification of expectedness of serious adverse reactions for purposes of SUSAR reporting.

The IMPD and SmPC will be stored in the site file. If it is necessary to update the IMPD and SmPC, the new version will be filed in the site file, old versions will be superseded, and the study team will be informed.

11.4 Preparation and labelling of Investigational Medicinal Product

The IMP will be prepared and labelled by Sharp Clinical Services UK. The process will be Annex 13 GMP compliant.

- Selenase 50mcg
  - De-blister Selenase 50mcg tablets.
  - Over-encapsulate one Selenase 50mcg tablet into DBAA el capsules with added Microcrystalline Cellulose
  - Count 93 Selenase 50mcg capsules into child-resistant tamper-evident containers and apply a randomised label to each container.
  - Pack two Selenase 50mcg containers, both with the same randomisation number, intocartons and apply the same randomised number label to each carton to produce patient packs and one QA pack

- Selenase 200mcg
  - De-blister Selenase 200mcg tablets.
  - Split one Selenase 200mcg tablet and over-encapsulate both halves into DBAA el capsules with added Microcrystalline Cellulose
  - Count 93 Selenase 200mcg capsules into child-resistant tamper-evident containers and apply a randomised label to each container.
  - Pack two Selenase 200mcg containers, both with the same randomisation number, into cartons and apply the same randomised number label to each carton to produce patient packs and one QA pack

- Placebo
    - ProduceDBAA el placebo capsules (filled with appropriate excipient)
    - Count 93 Placebo capsules into child-resistant tamper-evident containers and apply a randomised label to each container.
    - Pack two Placebo containers, both with the same randomisation number, into cartons and apply the same randomised number label to each carton to produce patient packs and one QA pack

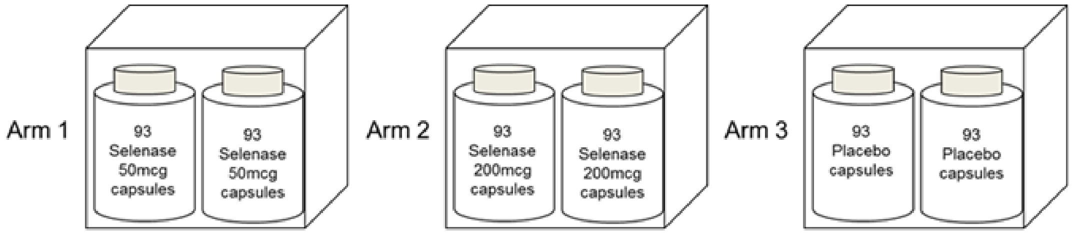

- Apply tamper evident seals to patient pack cartons.
- Take one patient pack per batch for QA destructive testing.
- Collate all patient packs together.
- Pack all patient packs into outers for shipping purposes.
- Carry out disintegration testing on the over-encapsulated Selenase tablets.
- Carry out QP final release.

11.5 Drug storage and supply

IMP will be provided by Sharp Clinical Services as above, specifically for use in this trial.

There are two planned manufacturing runs, to allow for possible changes to total participant number after the interim analysis. Further runs may be added if required based on the final sample size, recruitment time and shelf life of IMP.

Each manufacturing run will be delivered to Sheffield Teaching Hospitals pharmacy, under the supervision of the trial pharmacist.

The IMP will be stored at 15 to 25°C in STH pharmacy. Temperature logs will be maintained by STH pharmacy.

The first bottle of 93 tablets will be dispensed at the baseline visit, and the second bottle of 93 tablets at the 13 week visit. Bottles will be labelled with the study identifier (STH19102) and participant randomisation code.

IMP will be dispensed by pharmacy on receipt of a completed study-specific prescription, signed by the PI or delegated registered doctor.

STH pharmacy will retain dispensing records.

Unused or expired IMP at STH will be sent for hazardous waste disposal as per pharmacy SOPs; a record will be kept by pharmacy of what is sent for disposal and reported to the Sponsor if requested.

A 5% excess of patient packs will be manufactured in case a participant loses or damages their medication. If a replacement needs to be supplied, Sharp Clinical Services will advise which spare pack should be used, according to their copy of the randomisation schedule.

Participants will be advised to keep the IMP at room temperature.

11.6 Dosage schedules

Participants will receive one of three doses:

- Selenase 200mcg
- Selenase 50mcg
- Placebo

Orally, once daily, in the morning

The total duration of treatment will be 26 weeks.

11.7 Dosage modifications

There are no planned dose modifications.

If a participant develop diabetes (fasting glucose ≥7.0mmol/l) or thyroid dysfunction (TSH outside the reference range and determined as clinically significant by the investigator) during the study (identified on safety monitoring bloods, or a new diagnosis reported by participant at monthly contact), their treatment will be stopped. If a participant experiences other adverse effects thought to be related to IMP the PI will decide if that participant should stop treatment.

11.8 Known drug reactions and interaction with other therapies

Ascorbic acid; absorption of selenium possibly reduced by ascorbic acid (give at least 4 hours apart)

Eltrombopag: selenium possibly reduces absorption of eltrombopag (give at least 4 hours apart)

11.10 Assessment of compliance

Compliance will be assessed by asking at each contact whether participants have missed any doses of treatment. Participants will also be asked to return their study medication bottles for a pill count.

The number of reported missed doses and pill count will be recorded in the CRF.

Patients with less than 75% compliance will continue in the trial and ITT analysis, but will be excluded from per-protocol analysis.

Participants who are non-compliant with the safety monitoring at three months (do not attend or decline to have blood test) will be withdrawn from the trial.

11.11 NIMP

To ensure vitamin D sufficiency, all participants will be given a single bolus dose of colecalciferol 100,000IU at the screening visit. The colecalciferol will be bulk supplied by STH pharmacy to the Clinical Research Facility and stored in a locked drug cupboard, solely for use in this study. A stock list will be maintained with the stored supply. The colecalciferol will be prescribed by the PI or delegated doctor on a day case prescription chart, and administered by the doctor or specialist research nurse. The administration will be recorded on the day case prescription and in the participant case report form.

At the request of the REC committee that approved this trial, there will be an additional bottle of open label placebo capsules released from SHARP. This will be the ‘practice placebo’; volunteers will be shown the capsules during the screening visit and offered the opportunity to try taking a placebo capsule if they are concerned about their ability to swallow them. These placebo capsules will be released from SHARP to STH pharmacy and STH pharmacy will supply

the full bottle for storage in the Clinical Research Facility. A drug accountability log will be kept to track usage and disposal of each capsule. This will also be recorded on the participant case report form.

12. RISKS AND BENEFITS

We recognise that in moving from an association study to a clinical trial there is a risk that the trial will be negative. For example, in cross-sectional studies of the effect of a diet rich in fruit and vegetables it appeared there was a benefit to bone (30), but this wasn't confirmed in an RCT (31). However, because selenium supplementation could be an effective, inexpensive, and well-tolerated option for a group with unmet clinical need, we think that the proof of concept from our association study justifies moving forward to an RCT rather than repeated observational studies.

There have been RCTs of selenium in other disease areas so we already have some estimate of dose and safety. Studies have investigated the effect of selenium supplementation on a range of other health outcomes including cognition, immune function, cancer, cardiovascular function, diabetes, fertility and reproduction (8).

Selenium is used routinely for Graves' thyroid ophthalmopathy (GO) without side effects or effect on thyroid status at doses of 200 mcg/day for six months (32, 33). The UK GO guidelines recommend that selenium can be initiated by general practitioners or hospital general physicians, and no monitoring of selenium levels or other safety measures is required during the six month course (34).

We plan to administer selenium at a maximum dose of 200 mcg per day. The Institute of Medicine upper level for selenium is 400 mcg/day, which is 2.5 times below the No Observed Adverse Effect Level (35). Therefore the risk of adverse effects in this study is very low.

There is a published incident of selenium poisoning in 227 people due to mislabelling of a supplement product in the USA. The median dose of selenium was 41,585 mcg/day for 30 days. On questioning 201 people had symptoms of toxicity (nausea and vomiting, brittle nails, hair loss, joint pain, fatigue, irritability and garlic-like taste), 118 had sought medical advice for their symptoms, but only one patient required hospitalisation, and none died (36).
We will ask participants about symptoms of toxicity at each study contact.

We will measure serum selenium concentration at screening, baseline, three months and six months to evaluate the magnitude of effect of the study doses on serum selenium levels. The available data suggest that the optimum serum selenium concentration is 130 to 150 mcg/l, and nearly everyone in the UK has serum selenium concentrations below this range (8). The population range of serum selenium in the USA is 80 to 200 mcg/l. We expect that a dose of 200mcg/day will increase serum selenium by an average of 90mcg/l so the number of women whose serum selenium might increase above 200mcg/l is small.

Selenium has a role in thyroid function. There is a reported association of high blood selenium with type 2 diabetes, although this was not confirmed in a prospective study with doses of 100 to 300 mcg/day for 6 months (19). We will exclude volunteers with diabetes or thyroid dysfunction at screening, and measure thyroid function, fasting glucose and insulin at screening/baseline, three months and six months. We will withdraw participants who develop thyroid dysfunction or diabetes during the study. We will analyse between-group differences in change in thyroid function tests and blood glucose as a secondary endpoint.

The data on serum selenium concentration, glucose, thyroid function and any possible symptoms of toxicity will be reviewed by the DMEC.

The study procedures (DXA bone densitometry, blood and urine sampling and muscle function tests) carry negligible risks to the participants.

The radiation dose has been assessed by a Radiation Protection Supervisor and Clinical Radiation Expert.

13. STATISTICS

13.1 Power calculation

A 20% (10nmol BCE/mmolCr) decrease in urine NTX is a plausible and clinically significant effect size.

In a previous study, 200mcg daily for six months increased serum selenium by 90mcg/L (19).

In our observational study of serum selenium and bone turnover markers, the regression coefficient of serum selenium and NTX/Cr was -0.101 (9), so if the relationship were causative we would expect an increase in serum selenium of 90mcg/L to be associated with a reduction in urine NTX/Cr of 9 nmol BCE/mmolCr (approximately 20%).

In support of this estimate, we would not expect as large a change in bone turnover as would be seen with a potent anti-resorptive drug such as a bisphosphonate (about 60%), but it might be similar to a weaker anti-resorptive such as a SERM. In a study of six months' treatment with the SERM lasofoxifene (37), NTX decreased by 29%. We have also used this study as a model for the likely SD (12.5) and the correlation between NTX/Cr at baseline and six months (0.7).

A 20% decrease in NTX is clinically significant; a 20% decrease in NTX (about 1 standard deviation decrease) with bisphosphonate treatment is associated with a 30% decrease in incident vertebral fracture (38).

To achieve 90% power to detect this mean difference at the 2.5% (two-sided) level will require 21 patients per group. To allow for dropout, group imbalance and the secondary endpoint analyses we will recruit 33 patients per group with serum selenium below 120mcg/l (99 patients in total).

We will not exclude women with baseline serum selenium above 120mcg/l, because it is important that the results of this study are generalisable into practice, but it is possible that there will be no benefit in women who are selenium replete at baseline. Therefore we will recruit enough participants to ensure that at least 99 women with baseline serum selenium below 120mcg/l are randomised for the primary endpoint analysis. We will determine the total number required from an interim analysis of the distribution of baseline serum selenium in the first 40 women recruited. In our previous cross-sectional study, 80% of European women had serum selenium below 120mcg/l, so we expect that we will need to recruit 120 in total. If at the interim analysis, less than 60% of women recruited have serum selenium below 120 mcg/l, the study recruitment strategy will be reviewed and discussed with the funder to determine if the trial should proceed. Therefore the maximum sample size will be 165 (if 60% of women have serum selenium below 120mcg/l)

We will conduct a secondary analysis of all participants to determine whether baseline serum selenium is a determinant of bone turnover response.

The power calculation was done by Dr Richard Jacques, statistician at the Sheffield School of Health and Related Research (ScHaRR).

13.2 Analysis plan

A detailed statistical analysis plan (SAP) will be developed and finalised prior to the locking of the trial database in consultation with the trial steering committee.

We will conduct an intention-to-treat analysis (all randomised participants) and per-protocol analysis (completing participants with 75% compliance).

Primary endpoint:

Urine NTX at six months in postmenopausal osteopenic or osteoporotic women with baseline serum selenium below 120 mcg/l

Baseline data will be assessed for comparability between the treatment groups. NTX/Cr will be assessed visually for normality and if it is non-Normal a log-transform will be applied, with results reported as percentage difference between geometric means. Analysis of covariance (ANCOVA) will be used to compare mean NTX/Cr between the groups adjusting for baseline NTX/Cr measurements. The Hochberg procedure will be used to account for multiple comparisons; P-values are ordered from smallest to largest. If the largest P-Value is less than 0.05 then all null hypotheses can be rejected. If not, if the second P-value is less than 0.05/2, then all bar the largest can be rejected, and so on (SA Julious, An Introduction to Statistics in Early Phase Trials).

Secondary endpoints:

Urine NTX at three months: analysis as described for the primary endpoint

Serum selenium, SePP, serum CTX, PINP, osteocalcin, thyroid function, glucose and insulin at three and six months: analysis as described for the primary endpoint.

BMD by DXA at six months: analysis as described for the primary endpoint.

We will examine impact of baseline selenium levels on the NTX response to selenium supplementation by fitting a linear model with NTX at follow-up as the dependent variable and baseline selenium, baseline NTX and dose as independent variables. With n=66 (receiving active treatment) the minimum correlation that can be detected with 90% power and 5% significance level is 0.44.

Short physical performance battery, grip strength, hydroperoxidases, glutathione peroxidase activity, highly sensitive CRP, IL-6 analysis as described for the primary endpoint.

There is no planned interim analysis of outcome measures. There will be an interim analysis of baseline serum selenium to determine the final sample size.

14. STUDY SETTING

This is a single centre study.

It will be conducted in the Sheffield NIHR Clinical Research Facility at the Northern General Hospital, Sheffield. The CRF is a purpose-built facility, and contains all the resources needed for this study, including research specialist nurses, DXA and sample handling. The CRF has a high standard of governance and oversight. All studies are conducted as per organisational SOPs which are compliant with GCP and other regulatory requirements. Studies taking place in the CRF are subject to regular monitoring and audit.

Participants will be identified through Sheffield Metabolic Bone Centre, but also through primary care Patient Identification Centres.

15. ETHICAL CONSIDERATIONS

The study will require REC, MHRA, HRA and local R+D approval and no study procedures will be undertaken until all approvals are in place.

The study will be conducted according to GCP and ICH regulations, and the Medicines for Human Use (Clinical Trials) Regulations 2004.

Written informed consent will be obtained from all participants, supported by a detailed Participant Information Sheet. Data and samples will be managed in accordance with the Data Protection Act and Human Tissue Act requirements.

There is no standard care pathway for women with osteopenia or osteoporosis whose fracture risk is not yet high enough to qualify for treatment. Usual practice would be to advise on modifiable risk factors (such as dietary calcium intake and smoking) and consider repeat bone densitometry in a few years. Therefore this intervention will be in addition to usual care, and there is no ethical concern about using a placebo control.

Selenium has already been used at these doses and duration of treatment in clinical studies and in usual care of Graves' ophthalmopathy without adverse events.

The study intervention and procedures carry negligible risk to participants. The radiation dose for two DXA measurements of the spine and hip is 45.2microSv, equivalent to 7 days' natural background radiation.

Participants will be given travel reimbursement of £20 per visit, or provided with taxi transport for study visits. They will not receive any other payment for taking part in the study.

At the end of their study participation, the PI or delegated medical practitioner will write to the participants GP to inform them that their patient has completed the study, and give advice on any further recommended treatment and follow-up.

16. RECRUITMENT

A log will be kept of:

- Number of patient records assessed for pre-eligibility in Metabolic Bone Centre
- Number of patients approached from Metabolic Bone Centre and GP mailing
- Number of responders from these approaches
- Number of volunteers responding to adverts

- For responders not eligible on pre-eligibility check we will record reason for ineligibility

All volunteers who pass pre-eligibility checks and attend for consent and screening will be registered and allocated a study screening number. The screening number will be in the format COS001, COS002 etc.

16.1 Participant identification

Participants will be identified from four sources:

- AUBM volunteer database

  AUBM keeps a database of previous study participants, screen failures from previous studies and volunteers who have asked to be added to the database for future research opportunities. All participants have given consent to be approached about future studies.

  Potential participants will be sent an invitation letter and PIS, with a reply slip to request or decline further contact from the study team. Potential participants who do not return the reply slip will be contacted once by telephone to follow-up the invitation.

  On receipt of a positive reply, the study team will make contact with the volunteer to discuss the study, answer questions and confirm pre-eligibility according to the inclusion/exclusion criteria.

  Volunteers who are willing to participate and meet pre-eligibility criteria will be invited to a study visit for informed consent and screening.

- Metabolic Bone Centre, Northern General Hospital

  Fracture risk assessment reports will be screened for potentially eligible participants. This will include identifiable personal information, but will be done by the PI (who is a member of the clinical care team) or other member of the clinical care team.

Potential participants will be sent an invitation letter and PIS, with a reply slip to request or decline further contact from the study team. Patients who do not return the reply slip will be contacted once by telephone to follow-up the invitation.

On receipt of a positive reply, the study team will make contact with the patient to discuss the study, answer questions and confirm pre-eligibility according to the inclusion/exclusion criteria.

Patients who are willing to participate and meet pre-eligibility criteria will be invited to a study visit for informed consent and screening.

- Mailing from GP surgeries

GPs will be asked to send an invitation letter with reply slip to women age over 55 registered with their practice, and to screen the mailing list to remove anyone that it would be inappropriate to approach (eg dementia or current serious illness).

This will include identifiable personal information, but will be done by the clinical care team at the GP practice. The practice will be given a search strategy for this screening activity to ensure that screening is transparent and terms are defined.

On receipt of a positive reply, the study team will make contact with the patient to discuss the study, send a PIS and confirm pre-eligibility according to the inclusion/exclusion criteria. Patients who are willing to participate and meet pre-eligibility criteria will be invited to a study visit for informed consent and screening.

- Posters/email/website/word of mouth

Emails will be sent to hospital and university staff, through central IT services. The University has an established 'volunteers' mailing list. Posters will be displayed around the hospital and university, at patient and public events relevant to health and osteoporosis, and other public places such as GP surgeries and local businesses. Information on the study will be displayed on the hospital and university website.

The wording of all of these media will be submitted for REC and HRA approval.

On receipt of enquiries from these sources, the study team will make contact with the volunteer to discuss the study, send a PIS and confirm pre-eligibility according to the inclusion/exclusion criteria. Volunteers who are willing to participate and meet pre-eligibility criteria will be invited to a study visit for informed consent and screening.

Volunteers through any route will not be actively asked to pass on study information to friends or family, but if friends or family express interest in the study they will be advised to contact the study team who will follow the same procedure as for poster and email responders.

16.2 Screening requirements

Medical history for inclusion/exclusion criteria

BMD spine and hip by DXA (results within the last 6 months can be used)

Blood tests –for diabetes mellitus, thyroid dysfunction, bone profile

Volunteers will be shown the study capsules and offered the opportunity to try taking a placebo capsule if they are concerned about their ability to swallow the capsules.

Eligibility will be confirmed by the PI or delegated medical practitioner.

16.3 Consent

The PI retains overall responsibility for the informed consent of participants at their site and will ensure that any person delegated responsibility to participate in the informed consent process is duly authorised, trained and competent to participate according to the ethically approved protocol, principles of Good Clinical Practice (GCP) and Declaration of Helsinki.

Consent will be obtained by a doctor or specialist research nurse with appropriate training and experience assessed and delegated by the PI

This study will not include vulnerable participants or participants who lack capacity.

Informed consent will be obtained prior to the participant undergoing any trial procedures.

Each prospective participant will receive a participant information sheet giving details of the study and will have at least 24 hours to consider their participation in the study. All participants will also be given a verbal explanation of the information in the PIS and the opportunity to ask questions before the first visit, during the consent process and throughout the duration of the study. The consent process will follow the Sheffield CRF SOP for informed consent.

Where a participant is able to consent but later becomes incapacitated, they will be withdrawn from the study. Data obtained up to that point will be retained and used in analysis. This is specified in the PIS.

17. RANDOMISATION

Participants will be block randomised equally to the three treatment arms.

The randomisation list will be generated by Sharp Clinical Services. The list will be supplied to STH pharmacy and a copy retained by Sharp.

At randomisation, participants will be allocated a study ID number which corresponds to their randomised medication number (the ID number will be in the format SE???, where ??? is the randomised medication pack number).

The code list will be stored in STH pharmacy, in a sealed signed envelope under the supervision of the study pharmacist, or out-of-hours duty pharmacist. This envelope is signed when opened and resealed for audit purposes. If emergency access is required out of hours, the PI or if unavailable, another metabolic bone consultant can be contacted through STH switchboard. If they agree that the code need to be broken, the duty pharmacist will access the list.

18. BLINDING

This is a double-blind trial. The investigators, clinical study team, participants and analysing statistician will be blind to treatment allocation.

The active treatment will be overencapsulated and a matched placebo manufactured to maintain the blind.

The blind will not be broken until final database lock.

The study code will only be broken before database lock for medical or safety reasons e.g. in the case of a serious adverse event where it is necessary for the investigator or treating health care professional to know which treatment the patient is receiving before the participant can be treated. Subject always to clinical need, where possible, members of the research team will remain blinded.

If unblinding is required, a formal request will be made by the investigator/treating health care professional. If the person requiring the unblinding is a not a member of the investigating team, they should discuss the break with the investigator, unless there is a clinical emergency which requires immediate knowledge of the allocation.The PI will document the breaking of the code and the reasons for doing so on the CRF, in the site file and medical notes. It will also be documented in the final study report and statistical report. The Investigating team will notify the Sponsor in writing as soon as possible following the code break detailing the necessity of the code break. The written information will be disseminated to the Data Safety Monitoring Committee.

19. PHARMACOVIGILANCE

19.1 Reporting Period

The reporting period for adverse events will be from the date of informed consent until 28 days after the last administration of IMP. Any AEs that are ongoing at the close of the reporting period will be followed up until either resolved or stable.

19.2 Definitions

An Adverse Event (AE) is defined as any untoward medical occurrence in a subject during the course of the trial. Pre-existing conditions, although they will be recorded, will not be regarded as AEs unless they worsen significantly. This will be determined by PI or delegated doctor. Recognised side effects of the IMP are fully documented in the Summary of Product Characteristics of the IMP in question. All adverse events will be recorded in a Case Report Form and assessed for seriousness, expectedness and causality by PI or delegated doctor.

A Serious Adverse Event (SAE) will be defined as an AE which either

a) Results in death

b) Is life-threatening

c) Requires hospitalisation or prolongation of existing hospitalisation

d) Results in persistent disability or incapacity

e) Consists of a congenital abnormality or birth defect

Important medical events that may not be immediately life-threatening or result in death or hospitalisation but may jeopardise the patient or may require intervention to prevent one of the other outcomes listed in the definition above will also be considered serious.

The PI or delegated doctor will determine the seriousness of each adverse event as per these criteria.

19.3 Causality

The PI or delegated doctor will determine the causality of each adverse event as defined here:

Unrelated or unlikely: a clinical event including laboratory test abnormality with temporal relationship to trial treatment or IMP administration, that makes a causal relationship incompatible or for which other drugs, chemicals or disease provide a plausible explanation. This will be counted as “unrelated” for notification purposes.

Possible: a clinical event, including laboratory test abnormality, with temporal relationship to trial treatment or IMP administration which makes a causal relationship a reasonable possibility, but which could also be explained by other drugs, chemicals or concurrent disease. This will be counted as “related” for notification purposes.

Probable: a clinical event, including laboratory test abnormality, with temporal relationship to trial treatment or IMP administration which makes a causal relationship a reasonable possibility, and is unlikely to be due to other drugs, chemicals or concurrent disease. This will be counted as “related” for notification purposes.

Definite: a clinical event, including laboratory test abnormality, with temporal relationship to trial treatment or IMP administration which makes a causal relationship a reasonable possibility, and which can definitely not be attributed to other causes. This will be counted as “related” for notification purposes.

A SAE whose causal relationship to a study IMP is assessed by the Chief Investigator as “possible”, “probable” or “definite” will be considered ‘related’ and is an Adverse Drug Reaction.

Suspected Unexpected Serious Adverse Reactions (SUSARs)

A SUSAR is any event which qualifies as an SAE and meets the criteria of being judged as possibly, probably or definitely related to study IMP and has a nature and/or severity of which is not consistent with the information about the medicinal product in question as set out in the summary of product characteristics, investigator brochure or IMP dossier for that product (ie it is 'unexpected').

A SUSAR will require expedited reporting as per the Clinical Trials Regulations. All serious adverse events that fall or are suspected to fall within these criteria shall be treated as a SUSAR until deemed otherwise.

19.4 Responsibilities

The Principal Investigator will:

- Assess the event for seriousness, expectedness and relatedness to the study IMP as set out above
- Record all SAEs on standardised SAE forms and report them to the Sponsor by fax (STH R&D 0114 2265937) or email (sae@sth.nhs.uk) within 24 hours of the becoming aware of the event.
- Take appropriate medical action, which may include halting the trial and inform the Sponsor of such action

The Sponsor will:

- Report SUSARs which are fatal and life-threatening to the Competent Authority within 7 days.
- Report SUSARs which are not fatal and not life-threatening to the Competent Authority within 15 days.
- Within a further 8 days send any follow-up information and reports to the MHRA.
- Make any amendments as required to the study protocol and inform the ethics and regulatory authorities as required

20. WITHDRAWAL

Participants will be withdrawn at the participant's request, if they have a serious adverse reaction, if they develop diabetes or thyroid dysfunction, if they lose capacity, if they require treatment with medication on the prohibited list or if they develop any new medical problems which would render the study measurements invalid.

If IMP is stopped, and if the participant is willing, wherever possible they will be asked to continue to complete study assessments unless the PI judges this to be inappropriate due to the participant's health or personal circumstances. This decision will be documented in the CRF and site file, reported to the DMEC and included in the final study report.

21. SAMPLE STORAGE AND ANALYSIS

Biological samples collected from participants as part of this trial will be transported, stored, accessed and processed in accordance with national legislation relating to the use and storage of human tissue for research purposes and such activities shall at least meet the requirements as set out in the 2004 Human Tissue Act.

Blood and urine samples will be obtained and processed according to a study-specific sample handling SOP. This study-specific SOP will be reviewed and approved by Sponsor and stored in the site file.

Consent to use and store the samples will be obtained according to the Human Tissue Act 2004.

Safety bloods will be measured in real-time by Sheffield Teaching Hospitals clinical laboratories.

Other blood and urine samples will be stored frozen at -80°C until completion of all visits, and measured in batches. Samples will also be stored for later measurement of additional biochemical factors relevant to the aims of this study and the interpretation of the results, such as, but not limited to, other bone metabolism and anti-oxidant markers.

Some samples will be sent to collaborators at other centres for specialist measurements. Any samples transferred will be anonymised, and subject to a material transfer agreement. Any sample remaining after analysis at other centres will be returned to STH as per the terms of the material transfer agreement.

Samples will be stored in the STH HTA licensed biorepository and on completion of the study will be adopted by the South Yorkshire and North Derbyshire Musculoskeletal Biobank (REC ref 15/SC/0132). Additional tests relevant to and bone metabolism and musculoskeletal health may be carried out on these samples in the future. Any such additional testing will be reviewed and approved by the Biobank committee prior to testing. This is detailed in the participant information sheet and on the informed consent form.

22. DATA PROTECTION

Data will be kept secure according to the Data Protection Act 1998. All paper records will be stored in locked filing cabinets. DXA images will be stored on CD, identified by study number, initials and date of birth in locked filing cabinets. All electronic data will be identified by study number initials and date of birth and will be stored in a password protected database, on password protected computers (and encrypted laptops).

Participants will be asked for consent for their GP to be informed of their participation in the study.

All data reported will be fully anonymised.

Study documentation will be stored securely for 15 years after the study has been completed as per Sponsor SOPs.

23. GOVERNANCE AND MANAGEMENT

Sheffield Teaching Hospitals NHS Foundation Trust will be the study Sponsor. The study will be conducted according to appropriate Sponsor SOPs, Sheffield NIHR CRF SOPs, AUBM SOPs, study specific SOPs which will be reviewed by Sponsor and Good Clinical Practice. The study will be monitored by the STH Research Department Sponsor at regular intervals according to their standard procedures. In addition the project will be subject to internal monitoring to ensure quality of data, including source data verification and review of investigator site file essential documents, informed consent procedures, inclusion and exclusion criteria, patient clinical documentation, case report form and source data consistency and safety documentation and reporting to ensure compliance with legislation.

Study procedures will be carried out in the Sheffield NIHR Clinical Research Facility. All research personnel employed by the University of Sheffield have STH honorary contracts and research passports. The study will be carried out in compliance with the protocol and in accordance with the AUBM and CRF standard operating procedures. Any instances of non-compliance with the protocol will be reported to Sponsor and recorded in the Trial Master File according to SOPs.

Jennifer Walsh (CI) (University of Sheffield) will write the protocol and regulatory submissions, supervise study staff, confirm subject eligibility, provide clinical oversight (including AE assessment), and lead report and manuscript writing. She will be a member of the TMG and attend the TSC.

Richard Eastell (University of Sheffield). will provide expertise in clinical trial design, clinical oversight, supervise the biochemical measurements and contribute to data interpretation, report and manuscript writing. He will be a member of the TMG and attend the TSC.

Tom Hill (Newcastle University) will provide clinical nutrition expertise, supervise the dietary assessments and contribute to data interpretation, report and manuscript writing.

Graham Williams (Imperial College London) will provide expertise on the interpretation of thyroid function and anti-oxidant results, contribute to report and manuscript writing.

John Mathers (Newcastle University) will provide expertise in assessing selenium status. He will contribute to study design, data interpretation and manuscript writing.

Cindy Cooper (University of Sheffield) will advise on trial design and set-up, data interpretation and manuscript writing. She will be invited to attend the TSC.

Study staff will be supervised on a day-to-day basis by JW and RE, and attend the monthly CTIMP meeting. The nurse, co-ordinator and project manager are line managed through the Sheffield NIHR CRF.

Sheffield CTRU have advised on study management and statistics. Statistical support for this study is provided by the School of Health and Related Research (ScHaRR), Dr Richard Jacques.

Recruitment progress, adverse events and any relevant new information will be reviewed at the Sheffield AUBM monthly CTIMP management meeting. These meetings are minuted and the minutes pertaining to this study will be given to the TSC.

A Trial Steering Committee will be convened for the study, with an independent chair from Sheffield CTRU and representation from the Sheffield Lay Advisory Panel for Bone Research. The TSC will meet six monthly during the two-year project period, and reports from each meeting will be submitted to NIHR.

A DMEC will be convened with an independent chair and statistician. The DMEC will meet six monthly from the start of recruitment, to review study progress, recruitment and protocol adherence. A member of the investigator team will be identified who can supply the necessary unblinded data to the DMEC for their review whilst maintaining the blind with the rest of the investigator team. The committee will receive unblinded information on serum selenium, insulin, glucose and thyroid function, adverse events and any possible symptoms of selenium toxicity. The DMEC will report summary blinded data to the TSC, unless there are any concerns which require consideration of unblinded information.

If it is necessary for the protocol to be amended, the amendment and/or a new version of the study protocol will be notified to or approved by the ethics committee. If new information becomes available that may affect subject participation or safety on the study, revised patient information sheets will be prepared and approved by the ethics committee and Sponsor before subjects are provided with this new information and asked to re-consent.

24. TIMELINES

Milestone 1: REC, MHRA and Sheffield R+D approval: Nov 2016

Milestone 2: First patient recruited: Dec 2016

Milestone 3: Last patient recruited: Oct 2017

Milestone 4: All study visits complete: Apr 2018

Milestone 5: Biochemical measurements complete: Jun 2018

Milestone 6: Database checked and locked

Milestone 7: Statistical analysis complete: July 2018

Milestone 8: Report and manuscript complete: Sep 2018

End of trial

The end of trial will be completion of biochemical analysis (which will be the last data items, expected to be two months after the last patient last visit). The sponsor will notify the MHRA and main REC of the end of the trial within 90 days of its completion. An end of trial report will be submitted to EudraCT by the Sponsor.

25. PATIENT AND PUBLIC INVOLVEMENT

The Sheffield Lay Advisory Panel for Bone Research have reviewed this study. The Panel was established in 2009, and have made valuable contributions to these aspects of research in the Academic Unit of Bone Metabolism since then. They have received training in research methods, research governance and grant application processes. The Panel have presented their work at national PPI conferences such as INVOLVE. There are currently ten members. Their meetings are supported by a senior research nurse and an administrator.

The panel are consulted at the start of study development, and advise on the prioritisation of possible new research.

This study has been assessed by the Panel. These are their comments: 'The Lay Panel has read the brief outlining the research proposal, and had the opportunity to receive a presentation and ask questions about it. The Panel feels that this is worthwhile research focussing on a group of patients for whom bone strength is important. It feels that participation will be attractive to patients who will like the idea of receiving a nutritional supplement rather than a medication. The Panel would be pleased to support this application.'

They have reviewed the lay summary.

They review and contribute to the participant information documents and advise on recruitment strategy. The Panel will receive updates on study progress at their monthly meetings, where they have the opportunity to discuss the study with investigators and other members of the study team. The Lay Panel will be invited to send a representative to the TSC, and the minutes of the TSC will be available to the whole Lay Panel.

The start of the study will be publicised through the University of Sheffield, Sheffield Teaching Hospitals and National Osteoporosis Society, with contact details for any members of the public who would like further information on the study protocol or conduct.

On completion of the study the Lay Panel will write a lay summary of the results which will be sent to study participants, and publicised through University of Sheffield, Sheffield Teaching Hospitals and the National Osteoporosis Society. The Lay Panel will also be offered the opportunity to present the results at national PPI meetings.

REFERENCES

1. Moghadaszadeh B, Beggs AH. Selenoproteins and their impact on human health through diverse physiological pathways. Physiology (Bethesda). 2006;21:307-15.
2. Kohrle J, Jakob F, Contempre B, Dumont JE. Selenium, the thyroid, and the endocrine system. Endocrine reviews. 2005;26(7):944-84.
3. Hatfield DL, Tsuji PA, Carlson BA, Gladyshev VN. Selenium and selenocysteine: roles in cancer, health, and development. Trends in biochemical sciences. 2014;39(3):112-20.
4. Jakob F, Becker K, Paar E, Ebert-Duemig R, Schutze N. Expression and regulation of thioredoxin reductases and other selenoproteins in bone. Methods in enzymology. 2002;347:168-79.
5. Duntas LH. Selenium and inflammation: underlying anti-inflammatory mechanisms. Hormone and metabolic research = Hormon- und Stoffwechselforschung = Hormones et metabolisme. 2009;41(6):443-7.
6. Vaananen HK, Zhao H, Mulari M, Halleen JM. The cell biology of osteoclast function. Journal of cell science. 2000;113 (Pt 3):377-81.
7. Nutrition SACo. SACN position statement on selenium and health Department of Health. 2013;https://www.gov.uk/government/uploads/system/uploads/attachment_data/file/339431/SACN_Selenium_and_Health_2013.pdf.
8. Rayman MP. Selenium and human health. Lancet. 2012;379(9822):1256-68.
9. Hoeg A, Gogakos A, Murphy E, Mueller S, Kohrle J, Reid DM, et al. Bone turnover and bone mineral density are independently related to selenium status in healthy euthyroid postmenopausal women. The Journal of clinical endocrinology and metabolism. 2012;97(11):4061-70.
10. Lauretani F, Semba RD, Bandinelli S, Ray AL, Guralnik JM, Ferrucci L. Association of low plasma selenium concentrations with poor muscle strength in older community-dwelling adults: the InCHIANTI Study. The American journal of clinical nutrition. 2007;86(2):347-52.
11. Zhang Z, Zhang J, Xiao J. Selenoproteins and selenium status in bone physiology and pathology. Biochim Biophys Acta. 2014;1840(11):3246-56.
12. Manolagas SC. From Estrogen-Centric to Aging and Oxidative Stress: A Revised Perspective of the Pathogenesis of Osteoporosis. EndocrRev. 2010.
13. Cao JJ, Gregoire BR, Zeng H. Selenium deficiency decreases antioxidative capacity and is detrimental to bone microarchitecture in mice. The Journal of nutrition. 2012;142(8):1526-31.
14. Min Z, Zhao W, Zhong N, Guo Y, Sun M, Wang Q, et al. Abnormality of epiphyseal plate induced by selenium deficiency diet in two generation DA rats. APMIS. 2015;123(8):697-705.
15. Moreno-Reyes R, Egrise D, Neve J, Pasteels JL, Schoutens A. Selenium deficiency-induced growth retardation is associated with an impaired bone metabolism and osteopenia. Journal of bone and mineral research : the official journal of the American Society for Bone and Mineral Research. 2001;16(8):1556-63.
16. Jacobsen SJ, Goldberg J, Miles TP, Brody JA, Stiers W, Rimm AA. Regional variation in the incidence of hip fracture - US white women aged 65 years and older. JAMA. 1990;264:500-1.
17. Moreno-Reyes R, Suetens C, Mathieu F, Begaux F, Zhu D, Rivera MT, et al. Kashin-Beck osteoarthropathy in rural Tibet in relation to selenium and iodine status. The New England journal of medicine. 1998;339(16):1112-20.
18. Lauretani F, Semba RD, Bandinelli S, Ray AL, Ruggiero C, Cherubini A, et al. Low plasma selenium concentrations and mortality among older community-dwelling adults: the InCHIANTI Study. Aging clinical and experimental research. 2008;20(2):153-8.
19. Rayman MP, Blundell-Pound G, Pastor-Barriuso R, Guallar E, Steinbrenner H, Stranges S. A randomized trial of selenium supplementation and risk of type-2 diabetes, as assessed by plasma adiponectin. PloS one. 2012;7(9):e45269.
20. Eastell R, Hannon RA, Garnero P, Campbell MJ, Delmas PD. Relationship of early changes in bone resorption to the reduction in fracture risk with risedronate: review of statistical analysis. Journal of bone and mineral research : the official journal of the American Society for Bone and Mineral Research. 2007;22(11):1656-60.
21. Hurst R, Collings R, Harvey LJ, King M, Hooper L, Bouwman J, et al. EURRECA-Estimating selenium requirements for deriving dietary reference values. Crit Rev Food Sci Nutr. 2013;53(10):1077-96.
22. Guralnik JM, Simonsick EM, Ferrucci L, Glynn RJ, Berkman LF, Blazer DG, et al. A short physical performance battery assessing lower extremity function: association with self-reported disability and prediction of mortality and nursing home admission. J Gerontol. 1994;49(2):M85-94.

23. Minneci C, Mello AM, Mossello E, Baldasseroni S, Macchi L, Cipolletti S, et al. Comparative study of four physical performance measures as predictors of death, incident disability, and falls in unselected older persons: the insufficienza Cardiaca negli Anziani Residenti a Dicomano Study. Journal of the American Geriatrics Society. 2015;63(1):136-41.
24. Veronese N, Bolzetta F, Toffanello ED, Zambon S, De Rui M, Perissinotto E, et al. Association between Short Physical Performance Battery and falls in older people: the Progetto Veneto Anziani Study. Rejuvenation Res. 2014;17(3):276-84.
25. Hahn M, Conterato GM, Frizzo CP, Augusti PR, da Silva JC, Unfer TC, et al. Effects of bone disease and calcium supplementation on antioxidant enzymes in postmenopausal women. Clinical biochemistry. 2008;41(1-2):69-74.
26. Cervellati C, Bonaccorsi G, Cremonini E, Romani A, Fila E, Castaldini MC, et al. Oxidative stress and bone resorption interplay as a possible trigger for postmenopausal osteoporosis. BioMed research international. 2014;2014:569563.
27. Hoeflich J, Hollenbach B, Behrends T, Hoeg A, Stosnach H, Schomburg L. The choice of biomarkers determines the selenium status in young German vegans and vegetarians. The British journal of nutrition. 2010;104(11):1601-4.
28. Hollenbach B, Morgenthaler NG, Struck J, Alonso C, Bergmann A, Kohrle J, et al. New assay for the measurement of selenoprotein P as a sepsis biomarker from serum. J Trace Elem Med Biol. 2008;22(1):24-32.
29. Crosley LK, Bashir S, Nicol F, Arthur JR, Hesketh JE, Sneddon AA. The single-nucleotide polymorphism (GPX4c718t) in the glutathione peroxidase 4 gene influences endothelial cell function: interaction with selenium and fatty acids. Mol Nutr Food Res. 2013;57(12):2185-94.
30. Macdonald HM, New SA, Fraser WD, Campbell MK, Reid DM. Low dietary potassium intakes and high dietary estimates of net endogenous acid production are associated with low bone mineral density in premenopausal women and increased markers of bone resorption in postmenopausal women. The American journal of clinical nutrition. 2005;81(4):923-33.
31. Neville CE, Young IS, Gilchrist SE, McKinley MC, Gibson A, Edgar JD, et al. Effect of increased fruit and vegetable consumption on bone turnover in older adults: a randomised controlled trial. Osteoporosis international : a journal established as result of cooperation between the European Foundation for Osteoporosis and the National Osteoporosis Foundation of the USA. 2014;25(1):223-33.
32. Marcocci C, Marino M. Treatment of mild, moderate-to-severe and very severe Graves' orbitopathy. Best practice & research Clinical endocrinology & metabolism. 2012;26(3):325-37.
33. Marcocci C, Kahaly GJ, Krassas GE, Bartalena L, Prummel M, Stahl M, et al. Selenium and the course of mild Graves' orbitopathy. The New England journal of medicine. 2011;364(20):1920-31.
34. Perros P, Dayan CM, Dickinson AJ, Ezra D, Estcourt S, Foley P, et al. Management of patients with Graves' orbitopathy: initial assessment, management outside specialised centres and referral pathways. Clinical Medicine. 2015;15(2):173-8.
35. Institute of Medicine. Dietary reference intakes for vitamin C, vitamin E, selenium and carotenoids: National Academies Press; 2000.
36. MacFarquhar JK, Broussard DL, Melstrom P, Hutchinson R, Wolkin A, Martin C, et al. Acute selenium toxicity associated with a dietary supplement. Archives of internal medicine. 2010;170(3):256-61.
37. Rogers A, Glover SJ, Eastell R. A randomised, double-blinded, placebo-controlled, trial to determine the individual response in bone turnover markers to lasofoxifene therapy. Bone. 2009;45(6):1044-52.
38. Eastell R, Barton I, Hannon RA, Chines A, Garnero P, Delmas PD. Relationship of early changes in bone resorption to the reduction in fracture risk with risedronate. Journal of bone and mineral research : the official journal of the American Society for Bone and Mineral Research. 2003;18(6):1051-6.

APPENDIX 1 – Physical function tests

Short Physical Performance Battery (SPPB)

The SPPB score is calculated from three tests: chair stand, tandem balance and 8 feet walk. A score between 0 (worst performance) and 12 (best performance) is obtained by totalling the scores from the three tests. All of the procedures will be carried out with the participant wearing comfortable walking shoes with no or minimal heels to eliminate the effect of footwear on performance.

- Repeated Chair Stand Test

The repeated chair stand test measures the ability of the participant to stand from a chair without using their arms; reflecting the strength of their legs. The participant will be asked to sit on a chair with their feet placed on the floor, squarely in front of them and their knees flexed slightly greater than 90° so that their heels are closer to the chair than the back of their knees. The participant will be asked to fold their arms across their chest and to keep them folded throughout the test. The participant will be asked to stand and sit one time to determine their capability to continue to the main part of the test and to familiarise them with the procedure. The participant will be asked to stand and sit five times without stopping, as quickly as possible. The time taken for the participant to complete the 5 stand and sit cycles will be recorded to the nearest one hundredth of a second. If the participant is unable to complete the test this will be recorded, with the number of completed cycles, where applicable.

Walking aids must not be used during the test and the procedure will be carried out with the participant wearing comfortable walking shoes with no or minimal heels to eliminate the effect of footwear on performance.

Grading:

0 = unable

1 = >16.7 sec

2 = 16.6-13.7 sec

3 = 13.6-11.2 sec

4 = <11.1 sec

- Balance Testing

The participant will be asked to stand, unaided, with their feet in three different positions for a maximum of 10 seconds per position.

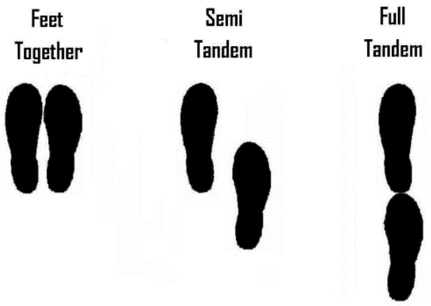

a) The first position is the feet in a side by side position. Participants can hold on to the researcher's arms in order to initially gain balance. When ready, the participant will be asked to hold the position, unaided, for a maximum of 10 seconds. Participants can move their arms, body and bend their knees to maintain balance but they should not move

their feet. If the participant is able to hold this position for 10 seconds then they may progress onto the next stand position.

b) The next stand position is a semi-tandem stand. The heel of one foot is placed by the big toe of another foot. Participants can hold on to the researcher's arms in order to initially gain balance. Participants will be asked to hold this position, unaided, for a maximum of 10 seconds. Participants can move their arms, body and bend their knees to maintain balance but they should not move their feet. If the participant is able to hold this position for 10 seconds then they progress onto the final stand position.

c) The final stand position is the full tandem stand. The participant will be asked to stand with the heel of one foot in front of and touching the toes of the other foot. Participants can hold on to the researcher's arms in order to initially gain balance. When ready, participants will be asked to hold this position, unaided, for a maximum of 10 seconds. Timing begins when the participant has let go of the researcher with their feet in the correct position. Participants can move their arms, body and bend their knees to maintain balance but they should not move their feet.

Balance Score:

0 = side by side 0-9 seconds or unable

1 = side by side 10 seconds, <10 seconds on semi-tandem

2 = semi-tandem 10 seconds, 0-2 seconds full tandem

3 = semi-tandem 10 seconds, 3-9 seconds full tandem

4 = tandem 10 seconds.

- 8 feet walk course (2.44 metres)

A 2.44 metres walk course will be marked out. Participants will be asked to walk at their usual pace from the start point to the other end of the course (2.44 metres). Participants will complete the test twice and an average time will be calculated.

Gait score:

0 = unable to complete

1 = >5.7 seconds

2 = 4.1-5.6seconds

3 = 3.2-4.0 seconds

4 = <3.1 seconds

Grip Strength

Hand grip strength will be measured using a digital hand dynamometer (Seahan Corp., Masan) to determine the strength of the hand and forearm muscles as an indicator of neuromuscular function. Participants will be seated, feet flat on the floor, and instructed to hold the dynamometer with their upper arm in line with their body, forearm at approximately 90º and their wrist un-rotated. Participants will be asked to grip the dynamometer as tightly as possible for five seconds and the result recorded. The test will be repeated three times on each hand, starting with

the right hand. At least thirty seconds rest will be given between each repetition. The maximal grip strength from the six measurements will be used for analysis.

APPENDIX 2 – Schedule of procedures

	Screening	Baseline	4W	8W	13W	17W	21W	26W	30W
Informed consent	X								
Medical history for eligibility	X								
DXA BMD*	X	X						X	
Colecalciferol 100,000 units	X								
Practice placebo (optional)	X								
Screening bloods[1]	X								
Blood for DNA		X							
Serum selenium		X			X			X	
Height and weight		X			X			X	
Pulse and blood pressure		X			X			X	
Bloods for end-of-study analysis[2]		X			X			X	
Urine for end-of-study analysis[3]		X			X			X	
Physical function tests		X			X			X	
Diet diary		X						X	
Concomitant medications		X	X	X	X	X	X	X	
Randomisation		X							
Dispensing of study drug		X			X				
Safety bloods[4]					X			X	
Compliance check			X	X	X	X	X	X	
Adverse events		X	X	X	X	X	X	X	X

*DXA will be done at screening for patients without BMD measurement in the last 6 months, and not repeated at baseline. For patients with BMD measurement within the last 6 months this measurement will be used for eligibility, and BMD will be measured at baseline.

1. Glucose, , HbA1c, TSH, bone profile, creatinine (non-fasted)
2. Selenium, selenoprotein P, PINP, OC, CTX, glutathione peroxidase, reactive oxygen species, hsCRP, IL-6, 25OH vitamin D
3. NTX/Cr
4. At baseline: glucose and insulin. At 13w and 26w: glucose, insulin, HbA1c, TSH
